# Dihydroxyacetone valorization with high atom efficiency via controlling radical oxidation pathways over natural mineral-inspired catalyst

**DOI:** 10.1038/s41467-021-27240-5

**Published:** 2021-11-25

**Authors:** Jinling Wang, Xingchao Dai, Hualin Wang, Honglai Liu, Jabor Rabeah, Angelika Brückner, Feng Shi, Ming Gong, Xuejing Yang

**Affiliations:** 1 National Engineering Laboratory for Industrial Wastewater Treatment, East China University of Science and Technology (ECUST), Shanghai, 200237 China; 2 State Key Laboratory of Chemical Engineering, ECUST, Shanghai, 200237 China; 3 Leibniz-Institut für Katalyse e.V. an der Universität Rostock (LIKAT), 18059 Rostock, Germany; 4 State Key Laboratory for Oxo Synthesis and Selective Oxidation, Lanzhou Institute of Chemical Physics, Chinese Academy of Sciences, Lanzhou, 730000 China; 5 Department of Chemistry, Fudan University, Shanghai, 200438 China

**Keywords:** Heterogeneous catalysis, Bioenergy, Catalytic mechanisms

## Abstract

Diminishing fossil fuel resources and calls for sustainability are driving the urgent need for efficient valorization of renewable resources with high atom efficiency. Inspired from the natural goethite mineral with Mn paragenesis, we develop cost-effective MnO_2_/goethite catalysts for the efficient valorization of dihydroxyacetone, an important biomass-based platform molecule, into value-added glycolic acid and formic acid with 83.2% and 93.4% yields. The DHA substrates first undergo C−C cleavage to selectively form glycolic acid and hydroxymethyl (·CH_2_OH) radicals, which are further oxidized into formic acid. The kinetic and isotopic labeling experiments reveal that the catalase-like activity of MnO_2_ turns the oxidative radicals into oxygen, which then switches towards a hydroxymethyl peroxide (HMOO) pathway for formic acid generation and prevents formic acid over-oxidation. This nature-inspired catalyst design not only significantly improves the carbon efficiency to 86.6%, but also enhances the oxygen atom utilization efficiency from 11.2% to 46.6%, indicating a promising biomass valorization process.

## Abstract (web summary)

Radicals-mediated Fenton chemistry has the potential for biomass valorization. Here, inspired from the natural goethite mineral, the authors develop a MnO_2_/goethite catalyst showing excellent yields of glycolic acid and formic acid from dihydroxyacetone, an important biomass-based platform molecule.

## Introduction

The global decline of fossil fuel resources and subsequent climate change issues have accelerated the need for renewable energy and sustainable resources. As a result, the co-production of chemicals with a high atom economy is being pursued with enthusiasm^1–6^. Biodiesel is a renewable bio-derived fuel with steadily increasing production over the years but has led to the over-supply of crude glycerol with relatively low utilization efficiency^7–11^. 1,3-dihydroxyacetone (DHA), which can be obtained via the catalytic oxidation or fermentation of glycerol and ketose^9,12^, is listed as one of the most important C3 platform molecules. DHA can be converted to C2 and C1 chemicals via selective C−C cleavage^9,13^, which enriches the product and value chain. For instance, selective C−C cleavage of DHA can simultaneously produce glycolic acid (GA) and formic acid (FA) with high atomic efficiency^10,14–16^, in which GA is an important intermediate for the synthesis of personal care products and FA is often used in the food and leather industry or as a source of formyl groups and a hydrogen storage carrier^17,18^.

Hydroxyl radicals (HO·) with the second strongest oxidative power (E^o^(OH/H_2_O) = 2.80 V/SHE)^19,20^, often generated by Fenton chemistry, have been proven as a powerful tool for the oxidative C−C cleavage of organic compounds, which guides the development of biomass upgrading, plastics degradation, wastewater treatment, and many other applications^21–24^. Recently, Cu/Al_2_O_3_ catalysts with isolated active sites were found to split H_2_O_2_ into HO· that selectively cleaves the C−C bonds in DHA to produce GA with excellent yields, and formamides and formates can be co-produced with GA efficiently when amines and alcohols are used as the co-substrates to stabilize and trap the FA intermediates^13^. However, this system still requires high H_2_O_2_ dosage (6 mmol H_2_O_2_ for 1 mmol DHA) to hedge against its inefficient decomposition leading to the low H_2_O_2_ utilization efficiency, while the high H_2_O_2_ dosage also induces the over-oxidation of products toward a low carbon efficiency. This dilemma is, in fact, a general problem in selective oxidation systems using H_2_O_2_ as an oxidant due to the transient kinetics and reactive nature of oxygen-centered radicals, which results in significant difficulties for H_2_O_2_-based oxidation with high atom efficiency^25,26^.

Compared to the C−C bond in alkane (83−90 kcal/mol), the C_α_−C bond in DHA is relatively weak (~80 kcal/mol for acetone)^27^, but is subject to keto-enol tautomerism^28,29^, which complicates the oxidative C−C cleavage of DHA. Under the attack of oxidative radicals, DHA could be oxidized to generate various organic radicals, making the controlled conversion to targeted chemicals difficult, while requiring a clear understanding of their precise fate and efficient regulation of radical pathways. For the present study, we were inspired from natural goethite minerals that are capable of cleaving the C−C bonds of organic compounds^30,31^. We succeeded in synthesizing a MnO_2_-attached goethite catalyst that can co-produce GA and FA with high yields. This system decreases the H_2_O_2_ stoichiometric ratio by a factor of 3 to achieve high atom efficiency, particularly with the highest oxygen atom efficiency ever reported. We found that the introduction of MnO_2_ can not only promote HO· generation and C−C cleavage, but also mediate the chain reaction to inhibit FA over-oxidation. We are convinced that our strategy of using nature-inspired abundant Fe/Mn-based catalysts to selectively oxidize biomass-derived molecules into value-added products can boost the future development of biomass valorization technologies significantly.

## Results

### Catalytic performance of iron-bearing minerals and natural goethite-inspired catalysts

**Fig. 1:**
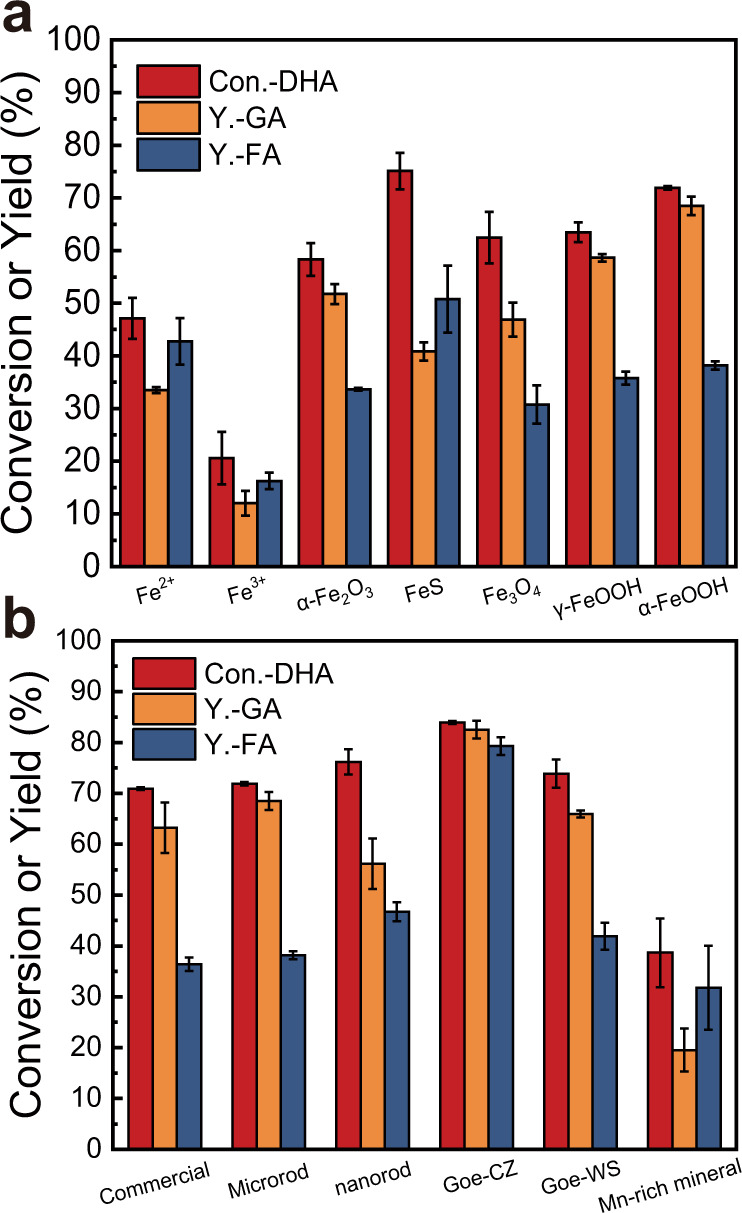
Catalytic performance screening of iron-based catalysts. **a** Catalytic activity for DHA oxidation over Fe-based ions and different Fe-containing minerals. **b** Catalytic activity for DHA oxidation over different goethite and Fe-containing Mn-rich minerals. The commercially available goethite (Sigma, 30−50 mesh) was used after grinding to more than 200 mesh. Reaction conditions: catalyst 30 μmol, DHA 1 mmol, H_2_O_2_ 0.2 mL (30 wt% H_2_O_2_ aqueous), 25 °C, 400 rpm, 24 h. Error bars represent standard deviations from triplicate experiments.

The typical iron-bearing minerals in the geochemical cycling of Fe, including hematite (α-Fe_2_O_3_), pyrite (FeS_2_), magnetite (Fe_3_O_4_), goethite (α-FeOOH) and lepidocrocite (γ-FeOOH), and the homogeneous Fenton catalysts (Fe^2+^ and Fe^3+^) were first screened for the selective oxidation of DHA. As shown in Fig. 1a, low DHA conversions of 20.0% and 47.1% were observed in the homogeneous Fe^3+^ and Fe^2+^ systems, respectively, indicating their weak activities of C−C bond cleavage. Although pyrite afforded the highest conversion of DHA (75.1%) among Fe-bearing minerals, it exhibited only moderate GA and FA yields and was almost completely dissolved after 24 h of reaction (the leaching ratio of Fe >90%, Supplementary Table 1). Taking into combined account of the DHA conversion and GA/FA yields, goethite outperformed other minerals with 71.9% DHA conversion, 68.5% GA yield, and 38.2% FA yield and maintained a satisfactory stability during the reaction. Further, we synthesized goethite with different sizes of microrods and nanorods to regulate its catalytic activity. Their corresponding structures were confirmed by SEM and XRD (Supplementary Fig. 1, 2). The conversion of DHA was improved from 71.9 to 76.2% when the size of goethite rods was reduced to nanoscale (Fig. 1b). Meanwhile, the GA yield decreased, indicating the difficulty of the size regulation towards high-yield GA and FA production under high DHA conversion.

**Fig. 2:**
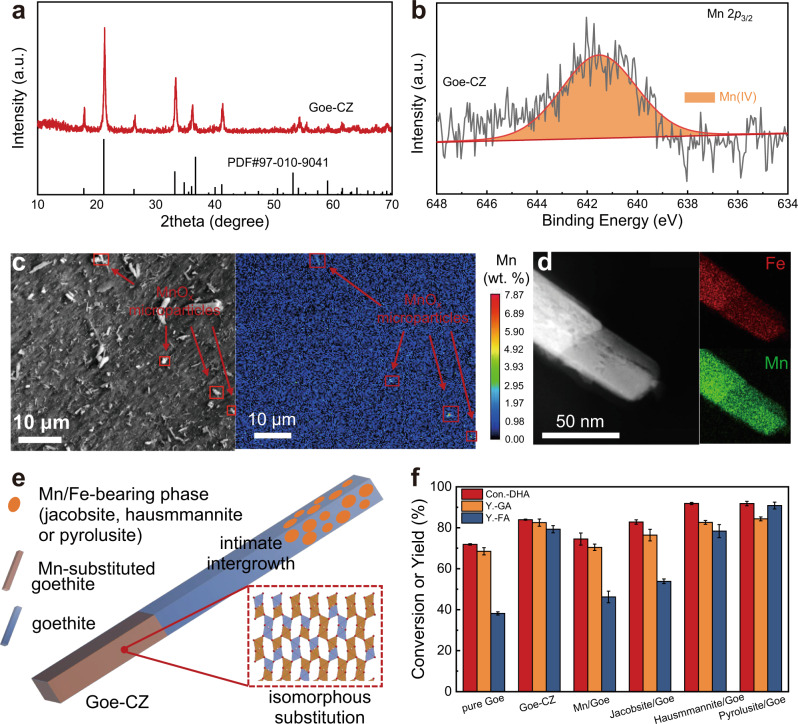
Structural and morphological characterizations of Goe-CZ and catalytic performance of mimic catalysts. **a** XRD pattern. **b** Mn 2*p*_3/2_ XPS spectra. **c** EPMA images (the spots with high Mn signal are proposed jacobsite (MnFe_2_O_4_), hausmmannite (Mn_3_O_4_), or pyrolusite (β-MnO_2_) microparticles). **d** STEM-EDS-mapping (Fe: red, Mn: green). **e** Schematic illustration of different distribution forms of Mn on Goe-CZ. **f** Catalytic activity for DHA oxidation over the mimic Mn/Fe-bearing mineral-attached and Mn-doped goethite catalysts. Reaction conditions: catalyst 30 μmol, DHA 1 mmol, H_2_O_2_ 0.2 mL (30 wt% H_2_O_2_ aqueous), 25 °C, 400 rpm, 24 h. Error bars represent standard deviations from triplicate experiments.

As the most thermodynamically stable and the most abundant Fe-bearing mineral^32^, goethite is widely found in soils of the temperate climate, marine and lake sediments and can occur in variable structures, such as intergrown Fe-/Mn-oxides, calcite and clay minerals or other atoms such as isomorphous crystal elements and trace metals (Al, Mn and etc.) can substitute for the Fe atoms^33–35^. To investigate these possible contributions of the unique natural structures, two natural goethite minerals, and a natural Mn-rich mineral were directly extracted from a mine and chosen for the selective oxidation of DHA. As shown in Fig. 1b, Goe-CZ, one of the natural goethites, showed a significant increase in GA and FA yield to 82.5 and 79.3% respectively (Fig. 1b). This serendipitous result impelled us to comprehensively characterize its composition and structure. Notably, the ICP analysis detected the presence of ~1.1 wt% of Mn in Goe-CZ, but not in any other goethites. In the natural environment, Mn can be substituted for Fe in the goethite structure by forming the isostructural groutite (α-MnOOH), nsutite (γ-MnO_2_), or ramsdellite (r-MnO_2_)^34,36^. With the increase of the Mn/Fe ratio, new separate Mn/Fe- or Mn-bearing phases, such as jacobsite (MnFe_2_O_4_), hausmannite (Mn_3_O_4_), or pyrolusite (β-MnO_2_), may appear together with goethite^36^. The Mn distribution in the Goe-CZ was further investigated due to its possible contribution to catalytic performance. No perceptible diffraction peaks of Mn-containing phases were observed in XRD patterns (Fig. 2a), but the nanocrystalline hausmannite and pyrolusite phases were successfully identified in the HRTEM images of the Goe-CZ sample (Supplementary Fig. 3). The XPS peaks at 641.5 eV can be attributed to Mn 2*p*_3/2_ signal of Mn(IV) species (Fig. 2b). The microscopic morphological analysis provided direct visual evidence for the proposed Mn distributions. SEM images revealed that the mineral is composed of 0.1−0.3 mm flake-like macroparticles, but microscopically forms a stacking of < 1 μm rod-like microparticles (Supplementary Fig. 4). According to the EPMA analysis with high spatial resolution (Fig. 2d), Mn was largely absent on most Goe-CZ surfaces, while a high Mn content was detected on some micron-sized particles, confirming the intergrowth of Mn-bearing phases with goethite. Besides, the Mn substitution into the goethite lattice was also observed in some particles, as indicating the overlap of Fe and Mn EDS signals at the rod edges (Fig. 2e).

As illustrated in Fig. 2f, the above characterizations clearly showed the two forms of Mn in the natural goethite (Goe-CZ), which inspired us to mimic the natural structure of Goe-CZ. We directly added the Mn nitrate salt to the precursors of goethite (Fe(NO)_3_) in the synthesis of goethite to mimic the substitution of isostructural groutite, nsutite, or ramsdellite in natural goethite, denoted as Mn-doped goethite (Mn/Goe). As shown in Supplementary Fig. 5, only the goethite phase was present in the Mn/Goe sample and the successful Mn doping in Mn/Goe was confirmed from ICP result (1.21% of Mn). The overlaid Mn 2*p*_3/2_ spectra of Goe-CZ and Mn/Goe showed a clear difference in the peak shapes (Supplementary Fig. 6). The Mn 2*p*_3/2_ signal of Mn/Goe was fitted to Mn(III) (641.5 eV) and Mn(IV) (639.9 eV) species (Supplementary Fig. 7). The intergrowth of Mn/Fe-bearing phases was mimicked by mechanical mixing the as-prepared jacobsite, commercial hausmannite, or pyrolusite with as-synthesized goethite microrods, denoted as jacobsite-attached goethite (jacobsite/Goe), hausmannite-attached goethite (hausmannite/Goe), and pyrolusite-attached goethite (pyrolusite/Goe), respectively. Similar to the Goe-CZ, there are no diffraction peaks assigned to Mn/Fe-bearing phases in any of the three samples (Supplementary Fig. 8), which should be ascribed to their low contents and high dispersion. Based on these results, we can conclude that the Mn distributions in natural Goe-CZ were largely reproduced by the doped and mixed samples, although there are still differences in the relative intensities of the diffraction peaks and the Mn valence states.

Selective oxidation of DHA was performed with the natural goethite-inspired mimic catalysts. Obviously, these catalysts with different Mn distributions showed significant differences in the catalytic activity. As shown in Fig. 2g, Mn/Goe showed only slightly improved yields compared to the pure goethite, while the three MnO_*x*_-attached goethites showed significant improvement in the catalytic performance, in which the GA/FA yields (84.3% for GA and 90.8% for FA) in MnO_2_/Goe even surpassed natural Goe-CZ. This indicates that the reaction benefits from β-MnO_2_ as a separate crystal phase. The control experiments without goethite exhibited lower GA and FA yields (Supplementary Fig. 9), which demonstrated the critical synergy between goethite and β-MnO_2_. By screening reaction parameters like reaction time and H_2_O_2_ concentration, we pinned down an optimal condition of 2 mmol of H_2_O_2_ for 24 h for the highest activity and selectivity (Supplementary Fig. 10). No obvious increase in GA and FA yields was observed under prolonged reaction time, and the GA and FA yields rose under relatively low H_2_O_2_ dosage but started to stagnate and even gradually decrease at high concentrations (>2.0 mmol) due to the over-oxidation of products. However, under O_2_-rich conditions (O_2_ = 1.2 MPa) the amount of H_2_O_2_ dosage could be further reduced to 1.8 mmol without a noticeable decrease in the GA and FA yield (Supplementary Fig. 11).

**Fig. 3:**
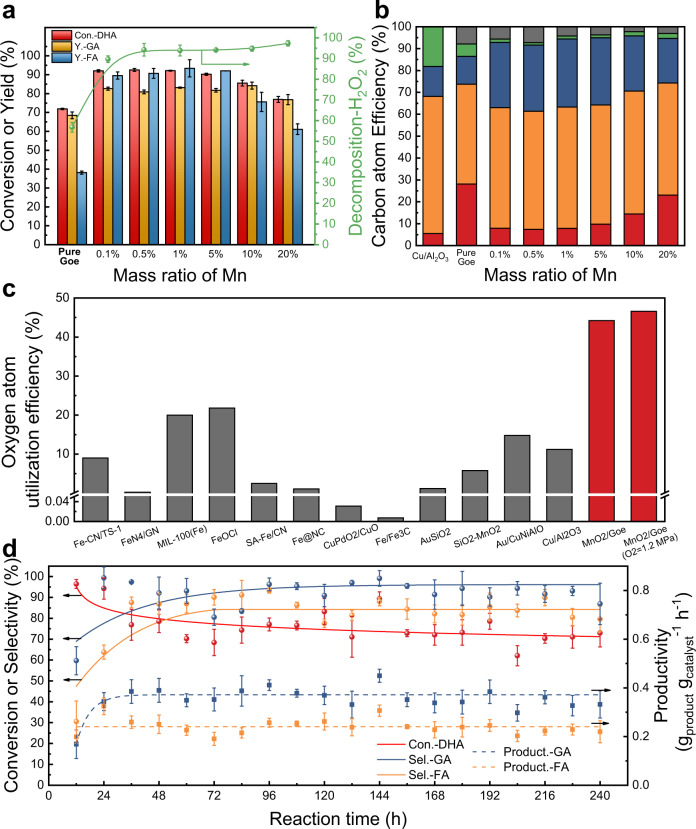
Catalytic activity, atom efficiency, and continuous stability test. **a, b** Catalytic activity and carbon atom efficiency for DHA oxidation over MnO_2_/Goe with different mass ratio of Mn. Red: unreacted DHA; Orange: GA; Blue: FA; Green: CO_2_; Gray: unknown intermediates. **c** Comparison of oxygen atom utilization efficiency for the typical heterogeneous catalytic oxidation processes using H_2_O_2_ as oxidant. The detailed information of these systems was listed in Supplementary Table 2. **d** Catalytic performance of the MnO_2_/Goe catalyst under the continuous flow condition. Reaction conditions for DHA oxidation: catalyst 30 μmol, DHA 1 mmol, H_2_O_2_ 0.2 mL (30 wt% H_2_O_2_ aqueous), 25 °C, 400 rpm, 24 h (batch experiment); catalyst 2 g, 1 mmol DHA in 2 mmol H_2_O_2_ aqueous solution (10 wt%), 0.15 mL/min, 25 °C (scale-up continuous experiment). Error bars represent standard deviations from triplicate experiments.

MnO_2_ particles profoundly impact on the DHA conversion, and their effect was then studied and optimized. The introduction of trace MnO_2_ (0.1 wt% of Mn in goethite) significantly improved the H_2_O_2_ decomposition and catalytic DHA conversion, but the reactivity remained nearly unchanged as the MnO_2_ ratio varied from 0.1 wt% to 5.0 wt% (Fig. 3a). The highest yield of GA and FA could reach 83.2 and 93.4% (Mn = 1.0 wt%), which outperformed the reported Cu/Al_2_O_3_ system by 52.4% with respect to FA yield^13^. The improved FA yield could lead to a carbon atom efficiency of 86.6−10.2% higher than the Cu/Al_2_O_3_ system—while the CO_2_ yield was drastically reduced from 18.2% in Cu/Al_2_O_3_ to 1.3% by avoiding the excessive oxidation of FA (Fig. 3b). This atom efficiency could be even higher due to a small percentage of unbalanced products (2.2−7.8%). In addition to carbon efficiency, the utilization of H_2_O_2_ was also drastically improved by using less than a third of the H_2_O_2_, reaching an overall atom efficiency of 67.2% compared to 19.4% in Cu/Al_2_O_3_ system. In particular, oxygen atom utilization of this catalyst impressively reached 44.2%, and could further increase to 46.6% when the amount of H_2_O_2_ dosage was reduced to 1.8 mmol under O_2_-rich conditions, which is at least two-fold higher than the highest heterogeneous oxidation processes using H_2_O_2_ as the oxidant (Fig. 3c and Supplementary Table 2)^13,26,37–46^. In industrial-scale chemical production that uses H_2_O_2_ as an oxidant, H_2_O_2_ cost is usually one of the main economic factors. Therefore, these chemical production processes are generally coupled with H_2_O_2_ synthesis plants, which are generally less expensive when considering the high production and transportation costs of commercial H_2_O_2_ (USD 0.43−2.87 kg^−1^ for production (27.5−50 wt% H_2_O_2_) and USD 2.87 km^−1^ for transportation)^47–49^. The significant reduction of H_2_O_2_ consumption in this system can indisputably save the cost of H_2_O_2_ plant construction and its corresponding maintenance, thereby increasing the overall economic efficiency of the process. In addition, our biomass-based process also includes carbon emission reduction potential, which is also may be economically advantageous from a sustainability point of view. When the percentage of MnO_2_ reached >5%, H_2_O_2_ decomposition dominated over the DHA conversion, decreasing yields of GA and FA. We also examined the effect of MnO_2_ on the homogeneous Fenton systems for DHA oxidation. However, the Fe^2+^ and Fe^3+^ systems only showed slightly higher DHA conversion and FA yield upon the addition of different MnO_2_ amounts, indicating that the homogeneous Fenton activity cannot be easily promoted by MnO_2_ (Supplementary Fig. 12). Further, we extended this system from DHA to other biomass-based platform molecules classified as: lignocellulose-derived compounds (glucose and xylose), lignin-based model compounds (β-O-4 ketone: 2-phenoxyacetophenone), and glycerol-based compounds (glycerol and 1,2-propanediol). Compared to the pure goethite, the MnO_2_/Goe catalyst showed improved conversion of the five substrates, especially for xylose, and achieved 16.39−37.78% enhancement in total carbon atom selectivity (Supplementary Fig. 13 and Supplementary Table 3). This result adequately demonstrates that this nature-inspired catalyst is effective not only for the selective oxidation of DHA but also for enhancing the C−C cleavage and suppressing the excessive product oxidation for other biomass-based substrates.

Although the reaction formed strongly acidic products, the rod-like morphology of the goethite composition in the catalyst remained intact after the catalytic reaction (Supplementary Fig. 14). During the reuse cycling experiments (Supplementary Table 4), the GA and FA yields decreased slightly from 83.2, 93.4 to 78.7%, 84.0% during the first cycle but remained nearly unchanged as the number of reuse cycles increased, indicating the satisfactory reusability of the MnO_2_/Goe catalyst. The leached Fe was lower than 0.14% in all five cycles and the leached Mn was in the range of 1.08−1.91% in the last four cycles, not including the first cycle. In order to investigate the activity and durability of the catalyst for practical industrial applications, we further tested its catalytic performance using scaled-up continuous (0.36 mol DHA/day) and batch (2.5 mol DHA, 2500-times scale-up) reactors. Within the 10-day continuous operation, the catalytic activity stabilized after the first day with similar GA and FA selectivity and slightly decreased DHA conversion due to the dilution of H_2_O_2_ required for safe operation under scaled-up conditions (Fig. 3d and Supplementary Fig. 15). The 2500× scaled-up batch system could also achieve a similar performance as that of the small-sized reactor system (Supplementary Table 5). As shown in Supplementary Table 6, the productivity (${{g}_{{{{{{\rm{product}}}}}}}\,{{g}_{{{{{{\rm{catalyst}}}}}}}^{-1}}\,{{{{{{{\rm{h}}}}}}}^{-1}}}$) of our scaled-up continuous and batch systems is much higher than that of the reported Cu/Al_2_O_3_ system, even comparable with some of the glycerol-based systems requiring high temperatures. These results clearly highlight the promising potentials of this catalyst for industrial applications.

### Understanding and improving the C−C cleavage of DHA

**Fig. 4:**
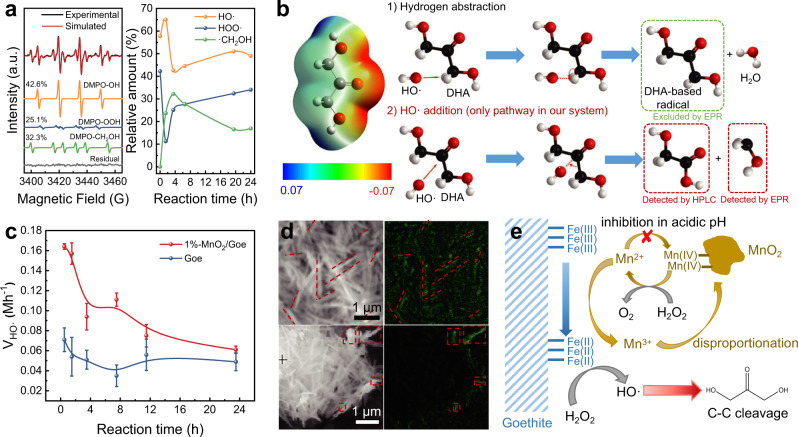
C−C bond cleavage behavior of DHA molecule. **a** EPR signals of the DMPO adducts (3.5 h) and relative amount of the DMPO-X adducts over time after adding DHA to the 1%-MnO_2_/Goe system (Hfs parameters: A_N_ = 15.1, A_H_ = 14.8 G for ·OH, A_N_ = 14.3, A_ßH_ = 11.4, A_γH_ = 1.2 G for ·OOH, and A_N_ = 16.0, A_H_ = 22.8 G for ·CH_2_OH). **b** Optimized structure and electrostatic potential distribution of DHA (the red surface corresponds to a negative region, whereas the blue surface corresponds to a positive region. Gray, red, and white spheres represent C, O, and H, respectively) and possible reaction pathways for the attack of HO· to DHA. **c** HO· formation rate over time in pure Goe and 1%-MnO_2_/Goe systems. Error bars represent standard deviations from triplicate experiments. **d** Scanning Auger mapping of Mn of fresh (bottom) and used (top) MnO_2_/Goe catalyst. **e** Scheme of the MnO_2_-involved HO· formation mechanism on goethite surface.

Activation of C−C bonds can be achieved in a variety of pathways including radical transfer, nucleophilic attack, and transition-metal insertion^50–52^. Since goethite can activate H_2_O_2_ to form radicals in a Fenton-like manner, we first used electron paramagnetic resonance (EPR) and the spin trap 5,5-Dimethyl-1-pyrroline N-oxide (DMPO) to monitor the formation of radicals during DHA selective oxidation. Typical EPR signals belonging to DMPO-OH and DMPO-OOH adducts were clearly resolved, suggesting the formation of HO· and HOO· radicals (Fig. 4a). According to the optimized structure and electrostatic potential distribution of DHA, the hydroxyl oxygen atoms are oriented towards ketyl oxygen atoms, which makes the DHA molecularly polarized and the attack of HO· more favorable along the opposite direction of oxygen atoms (Fig. 4b). There are mainly two routes of reacting DHA with HO· radicals: (1) hydrogen abstraction of the α-carbon by a HO· radical to form a DHA-based radical; (2) the addition of a HO· radical to the carbonyl group to form a carbonyl-centered radical, which can be then decomposed into GA and a hydroxymethyl radical (Fig. 4b). Although the formation of GA and FA implied that the latter pathway dominated the DHA oxidation in our system, we probed the radical intermediates with the aid of EPR. In addition to DMPO-OH and DMPO-OOH adducts, a new signal with hyperfine splitting parameters of A_N_ = 15.9 G and A_Hβ_ = 22.7 G could be assigned to the DMPO-hydroxymethyl radical adduct (DMPO-CH_2_OH), which indicated the DHA molecule was activated by the addition of HO· to carbonyl group. There was no signal of any other radicals observed, completely ruling out the hydrogen abstraction pathway. The relative amount of fitted DMPO-·CH_2_OH adducts over time showed a negative correlation with that of DMPO-·OH, suggesting a cause-and-effect relationship between the two radicals (Fig. 4a). The DMPO-·CH_2_OH adduct increased rapidly at the early stage and reached a peak at 4 h (30%), indicating that the C−C cleavage took place mainly in the early stages.

Moreover, the 1%-MnO_2_/Goe catalyst exhibited a HO· generation rate of 0.164 M·h^−1^ at the first hour, more than twice as high as that of goethite (0.071 Mh^−^^1^) (Fig. 4c). This enhancement progressively decayed over time, which agrees with the early-stage C−C cleavage processes. Unlike iron (hydro)oxides, MnO_2_ cannot activate H_2_O_2_ to generate HO·, but rather catalyzes the decomposition of H_2_O_2_ via a two-electron transfer to O_2_ (Eqs. 1–3)^53^. Therefore, the HO· enhancement is likely caused by the MnO_2_/Goe synergy. As we observed how leaching of Mn^2+^ and the acidic environment prohibited the cycling of reduced Mn^2+^ back to MnO_2_, we anticipated that the Mn^2+^ accumulation near the interface could potentially reduce the number of Fe(III) sites for promoting HO· generation due to the proximity of the two redox couples (Mn^3+^/Mn^2+^ = 0.9−1.5 V, goethite/Fe(II) = ~1.0 V at pH = 1) (Eq. 4)^54^. Such Mn^2+^ effect was then surveyed by the pyrophosphate (P_2_O_7_^4−^) complexing experiment. The presence of P_2_O_7_^4−^ ligand traps Mn^2+^ and prevents redox shuttling to goethite, which in turn greatly inhibits the conversion of DHA at 10 mM P_2_O_7_^4−^ (Supplementary Fig. 16a)^55,56^. Consistently, when Mn^2+^ ions were directly added to the pure goethite system, an activity increase was also observed (Supplementary Fig. 16b). Such redox cycle effect was also supported by the higher fraction of Fe(II) in the Fe 2*p*_3/2_ XPS for MnO_2_/Goe (Supplementary Fig. 16c and Supplementary Table 7).

During the Mn^2+^-promoted reduction of Fe(III), the generated Mn^3+^ returns to MnO_2_ via rapid disproportionation under the strongly acidic environment (Eq. 5)^57^, which was further verified by Auger mapping. As shown in Fig. 4e, Mn signals appeared on the surface of goethite and were distributed along the rod-like structure after the reaction compared to the fresh MnO_2_/Goe catalyst, which indicates that the Mn^2+^ oxidation and the following Mn^3+^ disproportionation had occurred on the goethite surface, forming MnO_2_ particles via Eqs. 4, 5^55^. Based on these results, we proposed the mechanism for the HO· generation catalyzed by the MnO_2_/Goe catalyst (Fig. 4f). The reductively released Mn^2+^ reacts with Fe(III) sites on the goethite surface to form Fe(II) sites. The formed Fe(II) sites can active H_2_O_2_ via a single-electron transfer pathway to produce HO· that cleave the C−C bonds of DHA (Eq. 6). Simultaneously, Mn^2+^ is oxidized to Mn^3+^ and then disproportionates back into MnO_2_ to recycle the catalyst.

1 $${{{{{{\rm{MnO}}}}}}}_{2}{+{{{{{\rm{H}}}}}}}_{2}{{{{{{\rm{O}}}}}}}_{2}{+{{{{{\rm{2H}}}}}}}^{+}\to {{{{{{\rm{Mn}}}}}}}^{2+}{+{{{{{\rm{2H}}}}}}}_{2}{{{{{{\rm{O}}}}}}+{{{{{\rm{O}}}}}}}_{2}$$

2 $${{{{{{\rm{Mn}}}}}}}^{2+}{+{{{{{\rm{2H}}}}}}}_{2}{{{{{{\rm{O}}}}}}}_{2}\to {{{{{{\rm{Mn}}}}}}({{{{{\rm{OH}}}}}})}_{2}{+{{{{{\rm{O}}}}}}}_{2}{+{{{{{\rm{2H}}}}}}}^{+}$$

3 $${{{{{{\rm{Mn}}}}}}({{{{{\rm{OH}}}}}})}_{2}+{{{{{{\rm{H}}}}}}}_{2}{{{{{{\rm{O}}}}}}}_{2}\to {{{{{{\rm{MnO}}}}}}}_{2}+{{{{{{\rm{2H}}}}}}}_{2}{{{{{\rm{O}}}}}}$$

4 $$\equiv {{{{{\rm{Fe}}}}}}({{{{{\rm{III}}}}}})+{{{{{{\rm{Mn}}}}}}}^{2+}\to \equiv {{{{{\rm{Fe}}}}}}({{{{{\rm{II}}}}}})+{{{{{{\rm{Mn}}}}}}}^{3+}$$

5 $${{{{{{\rm{2Mn}}}}}}}^{3+}+{{{{{{\rm{2H}}}}}}}_{2}{{{{{\rm{O}}}}}}\to {{{{{{\rm{MnO}}}}}}}_{2}+{{{{{{\rm{Mn}}}}}}}^{2+}+{{{{{{\rm{4H}}}}}}}^{+}$$

6 $$\equiv {{{{{\rm{Fe}}}}}}({{{{{\rm{II}}}}}})+{{{{{{\rm{H}}}}}}}_{2}{{{{{{\rm{O}}}}}}}_{2}+{{{{{{\rm{H}}}}}}}^{+}\to \,\equiv {{{{{\rm{Fe}}}}}}({{{{{\rm{III}}}}}})+{{{{{\rm{HO}}}}}}\cdot +{{{{{{\rm{H}}}}}}}_{2}{{{{{\rm{O}}}}}}$$

### Regulating the ·CH_2_OH transformation to FA

**Fig. 5:**
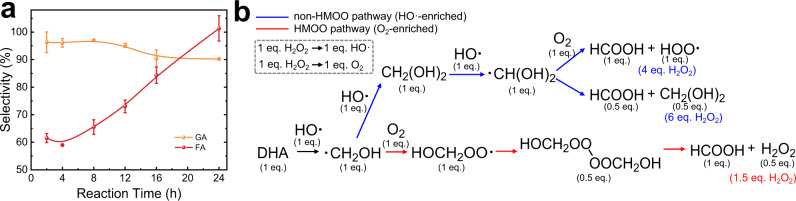
Products selectivity and reaction pathways analysis. **a** Selectivity profile over MnO_2_/Goe-1% catalyst as a function of time (the change in the concentrations of DHA, GA, and FA was shown in Supplementary Fig. 16). Error bars represent standard deviations from triplicate experiments. **b** Stoichiometric analysis of H_2_O_2_ required to produce 1 molar equivalent FA based on the different pathways.

To explore the chain propagation induced by the C−C cleavage, we first investigated the kinetics of GA and FA formation on the MnO_2_/Goe catalyst (Fig. 5a). The selectivity of GA remained essentially unchanged and only slightly decreased after 12 h due to the possible consecutive oxidation of GA. Notably, the selectivity of FA was relatively low at the early stage but exhibited an unusual trend of higher selectivity under longer reaction time. These results are consistent with the evolution of ·CH_2_OH species (Eq. 7), impelling us to investigate their reaction pathway.

7 $${{{{{{\rm{CH}}}}}}}_{2}{{{{{{\rm{OHCOCH}}}}}}}_{2}{{{{{\rm{OH}}}}}}+{{{{{\rm{HO}}}}}}\cdot \to {{{{{{\rm{CH}}}}}}}_{2}{{{{{\rm{OHCOOH}}}}}}+\cdot {{{{{{\rm{CH}}}}}}}_{2}{{{{{\rm{OH}}}}}}$$

As a radical, ·CH_2_OH can inevitably self-couple to form ethylene glycol. In our system, only traces of ethylene glycol were detected with very small production values (<0.1% DHA conversion) (Supplementary Fig. 18), indicating that the self-coupling of ·CH_2_OH radicals is not kinetically dominant. Alternatively, in the HO·-enriched Fenton system, ·CH_2_OH radicals can first combine with HO· to form hydrated formaldehyde (CH_2_(OH)_2_) (Eq. 8) and then be oxidized to ·CH(OH)_2_ radicals and FA (Eqs. 9–11)^58^. The schematic diagram based on elementary reactions provided a clear stoichiometric analysis (Fig. 5b), which turned out that converting 1 mole of DHA to FA via the HO·-enriched pathway requires 4 or 6 equivalents of H_2_O_2_. However, in our system, only 2 equivalents of H_2_O_2_ were added, indicating that other species rather than HO· should act as the oxidant of ·CH_2_OH radicals. In addition to HO·, O_2_ generated through H_2_O_2_ decomposition (Eq. 5) may also react with ·CH_2_OH radicals as another oxygen source. Hydroxymethyl peroxy radicals (·OOCH_2_OH, HMOO) can be generated under oxygen-rich environment (Eq. 12) and can subsequently undergo decomposition to form FA (Eq. 13)^59,60^. The stoichiometric analysis of this process in Fig. 5b suggests that the conversion of 1 mole of DHA to FA only requires 1.5 molar equivalents of H_2_O_2_, much lower than that of the non-HMOO pathway.

8 $$\cdot {{{{{{\rm{CH}}}}}}}_{2}{{{{{\rm{OH}}}}}}+{{{{{\rm{HO}}}}}}\cdot \to {{{{{{\rm{CH}}}}}}}_{2}{({{{{{\rm{OH}}}}}})}_{2}$$

9 $${{{{{{\rm{CH}}}}}}}_{2}{({{{{{\rm{OH}}}}}})}_{2}+{{{{{\rm{HO}}}}}}\cdot \to \cdot {{{{{{\rm{CH}}}}}}({{{{{\rm{OH}}}}}})}_{2}+{{{{{{\rm{H}}}}}}}_{2}{{{{{\rm{O}}}}}}$$

10 $$\cdot {{{{{{\rm{CH}}}}}}({{{{{\rm{OH}}}}}})}_{2}+{{{{{{\rm{O}}}}}}}_{2}\to {{{{{\rm{CHOOH}}}}}}+{{{{{\rm{HOO}}}}}}\cdot$$

11 $$2\cdot {{{{{{\rm{CH}}}}}}({{{{{\rm{OH}}}}}})}_{2}\to {{{{{\rm{CHOOH}}}}}}+{{{{{{\rm{CH}}}}}}}_{2}{({{{{{\rm{OH}}}}}})}_{2}$$

12 $$\cdot {{{{{{\rm{CH}}}}}}}_{2}{{{{{\rm{OH}}}}}}+{{{{{{\rm{O}}}}}}}_{2}\to {{{{{{\rm{HOCH}}}}}}}_{2}{{{{{\rm{OO}}}}}}\cdot$$

13 $${{{{{{\rm{2HOCH}}}}}}}_{2}{{{{{\rm{OO}}}}}}\cdot \to [{{{{{{\rm{HOCH}}}}}}}_{2}{{{{{\rm{OO}}}}}}-{{{{{{\rm{OOCH}}}}}}}_{2}{{{{{\rm{OH}}}}}}]\to {{{{{\rm{2CHOOH}}}}}}+{{{{{{\rm{H}}}}}}}_{2}{{{{{{\rm{O}}}}}}}_{2}$$

**Fig. 6:**
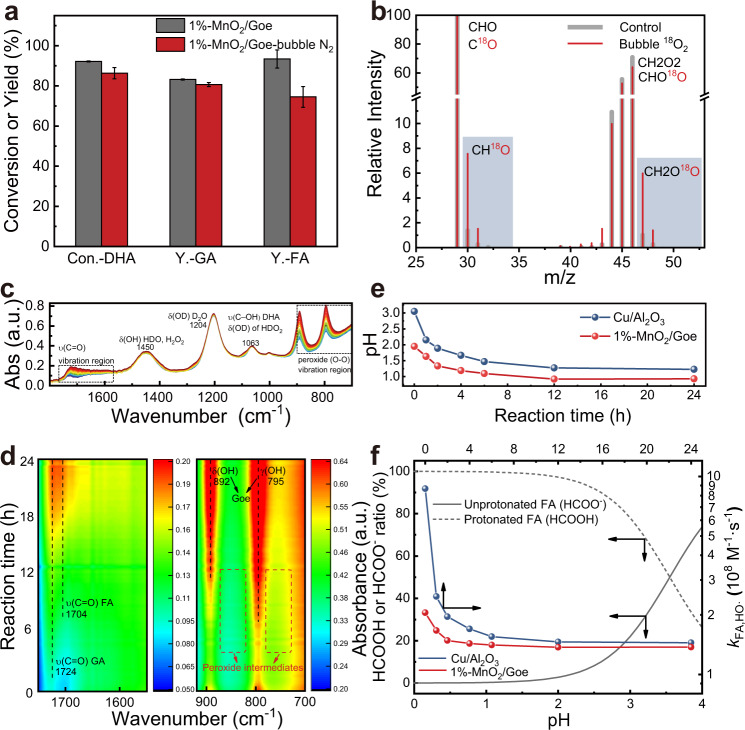
FA formation investigations. **a** Effect of bubbling N_2_ on the activity of MnO_2_/Goe catalyst. Error bars represent standard deviations from triplicate experiments. **b** MS signals of FA and its fragments during conversion of DHA with/without bubbling ^18^O_2_. **c**, **d** Operando ATR-FTIR spectra during conversion of DHA in D_2_O and the corresponding carbonyl bond and organic peroxide bond vibration regions. **e**, **f** Variation in pH during the reaction process using 1%-MnO_2_/Goe and Cu/Al_2_O_3_ catalysts and the corresponding protonated/unprotonated FA ratio with the change of pH value and the calculated average intrinsic rate constants of FA with HO·. The calculation of the ratio and average intrinsic rate constants were described in SI. The preparation and reaction conditions of Cu/Al_2_O_3_ were the same as that in the literature^13^.

Due to the catalase-like activity of MnO_2_, we discovered that the dissolved oxygen concentration was significantly higher in our MnO_2_/Goe system than in the pure Goe system (Supplementary Fig. 19), especially in the first few hours (31.8 ppm vs. 11.0 ppm). When we continuously bubbled N_2_ to remove dissolved oxygen during the reaction, the FA yield remarkably decreased (Fig. 6a), while the DHA conversion and GA yield were only slightly inhibited. Correspondingly, for the pure goethite system under O_2_ pressure, the FA yield increased significantly with increasing O_2_ pressure (Supplementary Fig. 20). These results suggested that O_2_ greatly contributed to the FA production as a reactant, implying the dominance of the HMOO path during ·CH_2_OH conversion. Since the presence of various radicals in the system hindered the identification of HMOO radicals, we designed ^18^O_2_ isotope experiments to validate their presence in our MnO_2_/Goe system. Once ^18^O_2_ molecules react with ·CH_2_OH radicals, ^18^O-labeled FA (HC^18^OOH, *m*/*z* = 47) should be generated, while only HC^16^O_2_H (*m*/*z* = 45) should be generated otherwise (Supplementary Fig. 21). As shown in Fig. 6b, the relative abundance of detected ^18^O-labeled FA and its fragments is enhanced by bubbling ^18^O_2_ into the reaction solution, confirming the existence of HMOO and its contribution to FA formation. These results indicate that the HMOO pathway provides an easily-accessed route to fix and utilize the O_2_ without involving the difficult O−O activation by spin inversion^61^.

Unlike short-lived HO· radicals (*t*_1/2_ = 10^−9^ s), HMOO radicals have longer life-time (*t*_1/2_ = 7 × 10^−5^ s) and tend to self-couple or couple with HOO· to form stable intermediates at low pHs^60,62^. As shown in Fig. 6c, Operando ATR-IR spectra provided insight into the generation and decomposition of products and intermediates of the DHA oxidation, particularly for ·CH_2_OH conversion. The bands at 1724 and 1704 cm^−^^1^ are assigned to the ν(C=O) vibration of GA and FA (Fig. 6d)^13^, and the evolution of the FA vibration band significantly lagged behind that of GA. Although the organic peroxidic vibration region (700−900 cm^−^^1^) was more disturbed by the O−H vibration of goethite, two weak bands at ~750 and ~850 cm^−1^ could still be observed during 4 h to 12 h, demonstrating the presence of peroxidic intermediates at this stage^63^. Correspondingly, it was found that about 30% of C1 products (FA, formaldehyde and hydrated formaldehyde) were lost during the first 2 h in our MnO_2_/Goe system. While this amount gradually decreased to close to zero after 12 h (Supplementary Fig. 22), the loss of unknown products might be attributed to the coupling intermediates of HMOO.

**Fig. 7:**
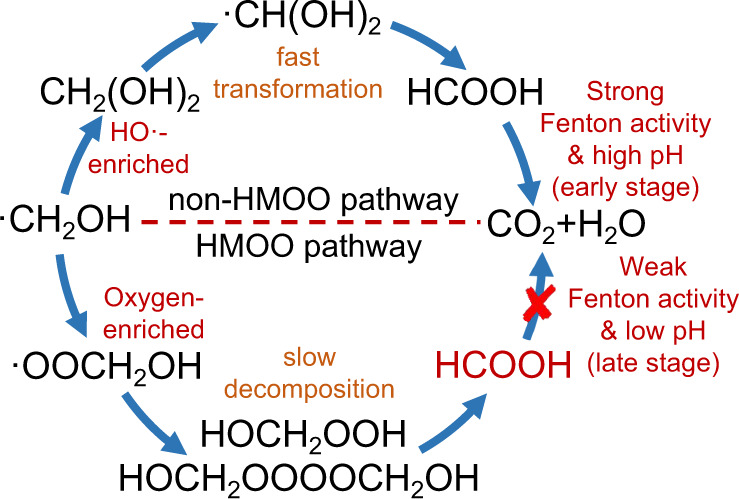
Schematic illustration of the pathway regulation. Scheme of the proposed HMOO and non-HMOO pathways for ·CH_2_OH transformation.

We further compared the kinetics of the HMOO and non-HMOO pathways (Supplementary Table 8). The persistent HMOO radicals and their coupling peroxidic intermediates confirmed the domination of the O_2_-rich step described in Eq.12, which reflects the slower kinetics of the HMOO pathway than that of the non-HMOO pathway. The slow transformation of ·CH_2_OH radicals to FA in the MnO_2_/Goe system bypasses the early-stage Fenton-like activity to avoid the excessive oxidation of FA. In addition, due to the continuous accumulation of FA, the pH of our MnO_2_/Goe system was maintained at a low range (0.9−1.6) throughout the reaction compared to that of the Cu/Al_2_O_3_ system (1.3−2.2) with low FA yield (Fig. 6e). The pH of the systems significantly affected the degree of protonation/deprotonation of FA, which changed the intrinsic rate constant of HO· with FA (${k}_{{{{{{{\rm{HCO}}}}}}}_{2}^{-},{{{{{{\rm{HO}}}}}}}\cdot }$ = 3.2 × 10^9^ M^−1^s^−1^ vs. ${k}_{{{{{{{\rm{HCO}}}}}_{2}{H}}},{{{{{{{\rm{HO}}}}}}}}\cdot }$=1.3 × 10^8^ M^−^^1^s^−^^1^)^64^. We calculated the ratio of protonated/deprotonated FA in the two systems and further determined the average intrinsic rate. The reaction rate in our MnO_2_/Goe system was always lower than that in the Cu/Al_2_O_3_ system; specifically, it was lower than one-fourth (2.0 × 10^8^ M^−1^ s^−1^ vs. 8.7 × 10^8^ M^−1^ s^−1^) in the early stage, which—in turn—greatly inhibited FA degradation (Fig. 6f). Based on these, a schematic comparison of the non-HMOO/HMOO pathways is shown in Fig. 7. Although the formed FA is inevitably degraded by HO· attack, the process is kinetically determined by two factors: the HO· concentration and the intrinsic rate constant of HO· with FA (Eq. 14). FA was rapidly generated but deeply oxidized in the early stage of the reaction under HO·-enriched environment. In contrast, the transformation of ·CH_2_OH to FA mediated by HMOO is a slow process, and, at low pH, is accompanied by the gradual release of FA to reduce the average intrinsic rate for FA oxidation. Consequently, this all together inhibits the FA degradation kinetics and regulates the·CH_2_OH transformation toward high FA selectivity.

14 $$-\frac{{{{{{\rm{d[FA]}}}}}}}{{{{{{\rm{d}}}}}}t}={k}_{{{{{{\rm{ins}}}}}}}\times {[{{{{{\rm{HO}}}}}}\cdot ]}\times {{{{{{\rm{[FA]}}}}}}}$$

## Discussion

Heterogeneous Fenton chemistry has shown increasing promise in industrial oxidation processes targeting high selectivity. However, one of the major obstacles for Fenton-based chemicals production is inefficient HO· generation. Inspired by natural goethite, we have successfully developed mimic MnO_2_/Goe catalyst via simple mechanical mixing for the co-production of GA and FA from DHA. The reactions proceed efficiently and selectively under room temperature and atmospheric pressure, with high carbon atom efficiency and high H_2_O_2_ utilization. EPR studied HO· generation kinetic and AEM images indicated that the incorporation of separate β-MnO_2_ phase can readily induce the acceleration of the Fe(III)/Fe(II) cycle on goethite surface to activate H_2_O_2_, which provides a tractable strategy for promoting Fenton-like activity without relying on sophisticated regulation in Fe active sites at the atomic level.

Understanding and regulating the evolution intermediates and radical is a more demanding challenge, which has rarely been achieved in heterogeneous catalysis. In this contribution, the stoichiometric analysis and kinetic experiments supported the switch of elemental steps towards FA formation with high H_2_O_2_ utilization efficiency, implying the contribution of the released O_2_ catalyzed by MnO_2_. Further, ^18^O isotopic labeling experiments and *operando* IR measurements confirmed the presence of hydroxymethyl peroxide (HMOO) radical that is an important indicator species for the proposed pathway and formed under O_2_-rich environment. These characterization and mechanistic studies depict an overall scenario of the C−C cleavage of DHA and the conversion from generated ·CH_2_OH radicals to FA, which demonstrates that—at least for HO·-dominated C−C cleavage—O_2_ can directly react with radical intermediates as a key oxygen donor to change the resulting product distribution.

In summary, this work not only develops a MnO_2_/Goe catalyst that can selectively oxidize DHA to co-produce GA/FA in excellent carbon/oxygen efficiency, but also provides a typical example of how to control the radical pathway during C−C cleavage. This system exhibits general feasibility for other biomass-based platforms although the detailed reaction mechanism needs to be elucidated, which highlights the great potential of Fenton chemistry as a powerful tool for the C−C cleavage and reveals an alternative route for the synthesis of various fine chemicals. It can also serve as a starting point for further studies on the interfacial reaction mechanism of radical-mediated C−C activation.

## Methods

### Chemicals and reagents

Fe(NO_3_)_3_·9H_2_O, Mn(NO_3_)_2_, sodium bicarbonate (NaHCO_3_), and NaOH were purchased from Aladdin Chemical Inc. (Shanghai, China). DHA, GA, FA, and 30 wt% H_2_O_2_ were obtained from Shanghai Titan Scientific Co., Ltd. (Shanghai, China). ^18^O_2_ (97 atom%) was purchased from Beijing InnoChem Scientific Co., Ltd. (Beijing, China). DMPO was purchased from Tokyo Chemical Industry Co., Ltd. (Tokyo, Japan). The chemicals used to synthesize goethite (α-FeOOH) are ACS grade and other chemicals are analytical grade. All chemicals were used without further purification. Milli-Q water was used throughout this study.

### Iron and manganese-containing minerals

Goethite, hematite (α-Fe_2_O_3_), magnetite (Fe_3_O_4_) were purchased from Sigma-Aldrich (St. Louis, MO, USA). Lepidocrocite (γ-FeOOH) was purchased from Alfa-Aesar (Haverhill, MA, USA). Hausmannite (Mn_3_O_4_) and pyrolusite (β-MnO_2_) were purchased from Shanghai Macklin Biochemical Co., Ltd. (Shanghai, China). Microrods, nanorods, Mn-doped goethite, and jacobsite (MnFe_2_O_4_) were synthesized according to procedures previously described (Supplementary Methods)^65–68^. Pyrite used in the study was obtained by directly mining from the commercial pyrite deposits located in Lincang County, Yunnan Province, China. Mn-rich mineral used in the study was obtained by directly mining from the commercial Mn oxides deposits located in Shijiazhuang City, Hebei Province, China, and has been identified as consisting of SiO_2_ and three Mn-containing phases (Todorokite, Groutite, and Birneesite) (Supplementary Fig. 23), but also contains a small amount of Fe (7.4 wt%). Two natural goethite minerals used in the study were obtained by directly mining from the commercial massive sulfide lead−zinc deposits located in Chenzhou (CZ) County, Hunan Province, China, and Wenshan (WS) County, Yunnan Province, China, were labeled as Goe-CZ and Goe-WS, respectively, in which they were both formed in the unsaturated oxidation zone of the deposits as the secondary minerals. MnO_*x*_-attached goethite was prepared by mechanical mixing using a tube roller mixer (MSK-MIX-T8, HF-Kejing, China) under the scroll mode (30 rpm) for 1 h. The commercially available goethite (30−50 mesh), natural goethite (>10 cm), and natural pyrite (1−5 cm) were grinded and sieved manually to above 200 mesh for the activity test, while other catalyst powders (>200 mesh) were directly used in the reaction. The structural information of all Fe/Mn-containing minerals is listed in Supplementary Table 9.

### Characterization methods

The morphology of the catalyst was characterized by scanning electron microscopy (SEM, ZEISS GeminiSEM 500, German). The manganese distribution in natural goethite was identified using electron probe micro analyzer (EPMA, JEOL JXA-8530F, Japan) and transmission electron microscope (TEM, Thermo Scientific Talos F200X, USA) equipped with high angle annular dark field (HAADF) and energy dispersive X-ray (EDX) analysis detectors. X-ray diffraction (XRD) patterns were collected on a Bruker D8 advance X-ray diffractometer (Bruker, USA) with a Cu Kα radiation (=1.5406 Å). The BET surface area was measured by N_2_ adsorption isotherms at 77 K using ASAP 2020 automated surface area analyzer (Micromeritics, USA). X-ray photoelectron spectroscopy (XPS) was collected on an ESCALAB 250Xi spectrometer (Thermo Scientific, USA) with a monochromatic Al Kα source (1486.6 eV, passing energy 20 eV), and all binding energies were calibrated with the C 1 s peak at 284.8 eV. Scanning Auger mapping (SAM) analysis was carried out using a PHI-710 Auger spectrometer (Physical Electronics, Inc., USA). Operando infrared absorption spectroscopy in the attenuated total reflection mode (ATR-IR) was collected on a ReactIR15 IR spectrometer (Mettler Toledo, USA). The formation of free radicals was examined by electron paramagnetic resonance spectroscopy (EPR, Bruker ELEXSYS 500-10/12, USA) using DMPO as a spin trap agent. The detailed ATR-IR measurements and DMPO-trapping procedures are described in Supplementary Methods.

### Computational methods

All computational procedure was carried out using Gaussian 09 and Gaussian view 6.0 programs^69^. The optimization structure and electrostatic potential calculation of DHA have been performed using the B3LYP functional and the 6−31 g* basis set.

### Selective C−C bond cleavage of DHA

The selective C−C bond cleavage of DHA was studied by batch experiments. In a typical experiment, 30.0 μmol iron (hydr)oxide, 1.0 mmol DHA, and 2 mmol H_2_O_2_ (30 wt% H_2_O_2_ aqueous solution) were added to a 5 mL glass reactor and reacted under a stirring speed of 400 rpm at 25 °C for 24 h. For the N_2_/^18^O_2_ bubbling experiment, the glass reactor was replaced with a modified HPLC sample vial containing a micro insert tube (300 μL) to reduce the FA loss and total evaporation loss. At a set time interval, the suspension sample was withdrawn and immediately filtered (0.22 μm PES) to remove the solids. The concentrations of DHA, GA, and FA were analyzed using a high-performance liquid chromatography (HPLC, Waters 2695, USA) equipped with a diode array detector (DAD, Waters 2996, USA). The total concentrations of formaldehyde and hydrated formaldehyde (methanediol) were analyzed by HPLC after 2,4-dinitrophenylhydrazine (2,4-DNPH) derivatization^70^. H_2_O_2_ concentration was analyzed spectrophotometrically using the titanium sulfate method^71^. The amount of generated CO_2_ was determined by GC-MS (Agilent 7890B, USA) with N_2_ in air as an internal standard. Dissolved oxygen (DO) was determined by a DO meter (INESA JPB-607A, China) in an enlarged system (10 mmol DHA, reaction solution volume of ~2 mL) due to the large size of the electrode head (1 cm in diameter). During the test, the DO electrode was first immersed 0.5 cm below the surface of the solution, and then the DO value was recorded after 30 s of stabilization. The relative abundance of FA, ^18^O-labeled FA, and their fragments were detected determined by gas chromatography–mass spectrometry (GC-MS, Shimadzu GCMS-QP2010Plus, Japan) with DB-FFAP column. The concentration of HO· was quantified by a chemical probe method using formic acid as the probe molecule^31^. More details are presented in Supplementary Methods.

### Reusability of MnO_2_/Goe catalyst

The catalysts after reaction were filtered using 0.22 μm PES membrane, washed with deionized water and dried overnight at 60 °C in a vacuum oven for the next use. To offset the unavoidable catalyst mass loss during the recovery (~20−40% for each run), we performed 10 sets of parallel experiments at the first cycle to recover enough catalyst for subsequent cycles. The leaching percent is defined as the molar ratio between the loss of metal and initially, added metal component.

### Scale-up of catalytic processes

For the continuous activity test, the reaction was carried out in a double-jacket tubular packed bed having dimensions of 100 × 1.5 cm (height × diameter) (Supplementary Fig. 24), with the upflow of liquid through the packed layer of particles (mixture of 16 mesh silicon carbide with 2.0 g 1%-MnO_2_/Goe catalyst). The mixture of 1 mmol DHA in 2 mmol H_2_O_2_ aqueous solution diluted to 10 wt% was pumped to the packed bed with a flow rate of 0.15 mL/min (360 mmol DHA/day) and the reaction temperature was maintained at 25 °C. The batch reaction was scaled up 2500 times in equal condition of the original system and was carried out in a 1 L double-jacket kettle (catalyst 75 mol, DHA 2.5 mol, 500 mL 30 wt% H_2_O_2_ aqueous, 25 °C, 400 rpm, 24 h) (Supplementary Fig. 25).

## Supplementary information

Supplementary Information

## Data Availability

The data that support the findings of this study are available from the corresponding author upon reasonable request.
