# Supplementary material for: Dihydroxyacetone valorization with high atom efficiency via controlling radical oxidation pathways over natural mineral-inspired catalyst
Source: Nat Commun. 2021 Nov 25;12:6840. doi: 10.1038/s41467-021-27240-5 (PMC8617048; doi:10.1038/s41467-021-27240-5)
Supplement: Supplementary file 1 — Supplementary Information [file 41467_2021_27240_MOESM1_ESM.pdf]

Supplementary Information

Dihydroxyacetone valorization with high atom efficiency via controlling radical oxidation pathways over natural mineral-inspired catalyst

Jinling Wang[+], Xingchao Dai[+], Hualin Wang, Honglai Liu, Jabor Rabeah, Angelika Brückner[*] Feng Shi, Ming Gong and Xuejing Yang[*]

Supplementary Methods

Synthesis of microrods of pure goethite. The synthesis method was adapted from Schwertmann and Cornell.[1] 40.4 g of $Fe(NO_3)_3 \cdot 9H_2O$ and 100 mL of Milli-Q H_2O were mixed in a 2 L polyethylene bottle. With stirring, 180 mL of 5 M KOH was added to this bottle and the solution changed from bright orange to red-brown precipitates at once. The suspension was diluted to 2 L with Milli-Q H_2O immediately and then was heated at 70°C in a water bath for 60 h. During the heating stage, the red-brown precipitates were gradually converted yellow-brown and settled to the bottom quarter of the bottle. The supernatant was discarded and the remaining suspension was filtered and washed with Milli-Q H_2O until pH<8. The resulting solid was dried at 353K.

Synthesis of nanorods of pure goethite. The synthesis method was adapted from Anschutz and Penn.[2] With stirring, 250 mL of 0.48 M $NaHCO_3$ was pumped to a 250 mL of 0.40 M $Fe(NO_3)_3 \cdot 9H_2O$ at a rate of 4.6 mL/min. During the pumped transfer, the solution changed from bright orange to dark-brown without obvious precipitation. The suspension was poured into a PTFE bottle and heated to boiling at 180°C in an oil bath, shaking every 40s. The boiling suspension was immediately plunged into an ice bath until it reached room temperature. The cooled suspensions were placed into dialysis bags (MWCO=2000), and then the dialysis bags were soaked into Milli-Q H_2O for 3 days, where the water was changed two times per day. After dialysis, the suspension was removed to a 500 mL PTFE bottle and the pH of the suspension was quickly adjusted to 12 using 5 M NaOH, where the color of the suspension had become darker. The suspension was heated at 90°C in an oil bath for 1 day, after which a dark yellow precipitate had settled to the bottom quarter of the bottle. The supernatant was discarded and the remaining suspension was treated following the same procedures as microrods of goethite.

Synthesis of microrods of Mn-doped goethite. The synthesis method was adapted from Gerth.[3] 100 mL of 1 M $Fe(NO_3)_3 \cdot 9H_2O$ was mixed with a certain amount of 0.5 M $Mn(NO_3)_2$. Then with stirring, the pH of the mixture was titrated to 4.5 using 1 M NaOH. After adding NaOH, the solution gradually changed from bright orange to dark red as the colloid precipitated, where the color deepened with the increase of Mn

content. The resulting precipitates were filtered and washed with Milli-Q H_2O until pH<8. The washed precipitates were transferred to a 1 L polyethylene bottle and then poured 1L 0.5 M NaOH into the bottle. The bottle was allowed to stand at room temperature for 1 day and heated at 70°C in an oil bath for 7 days and shaken up once per day. During the heating stage, the precipitates were gradually converted brown and settled to the bottom quarter of the bottle. The supernatant was discarded and the remaining suspension was treated following the same procedures as microrods of goethite.

Synthesis of jacobsite. The synthesis method was adapted from Hu and Chen.[4] 200 mL of 0.1 M $Mn(NO_3)_2$ and 0.2 M $Fe(NO_3)_3 \cdot 9H_2O$ was first prepared using deoxygenated Milli-Q H_2O with vigorous stirring. Then, while stirring, the pH of the above mixture was titrated to 11 using 2 M NaOH. After adding NaOH, the solution gradually changed from brownish yellow to black precipitates. The mixture was then heated to 100°C and kept at this temperature for 2 hours. The resulting precipitates were filtered and then treated following the same procedures as microrods of goethite.

HPLC analysis. The concentrations of DHA, glucose, xylose, glycerol, 1,2-propanediol, and all organic acidic products were analyzed using HPLC-DAD (HPLC-RID for glucose, xylose, glycerol and 1,2-propanediol) equipped with an Aminex HPX-87H (Bio-mad) column. The eluent was 5 mM H_2SO_4 with a flow rate of 0.5 mL/min and the temperature of the column was kept at 65°C。The sample was diluted 20 times with Milli-Q H_2O before injection.

2-phenoxyacetophenone, benzoic acid, catechol and phenol were analyzed using HPLC-DAD equipped with a Zorbax Eclipse XDB-C18 (Agilent) column. The eluent was a mixture of methanol and water (65/35, v/v) with a flow rate of 1.0 mL/min and the temperature of the column was kept at 30°C.

Operando ATR-IR measurements. Operando ATR-IR spectra were recorded in the range from 650 to 1900 cm^{-1} by a Mettler Toledo *ReactIR15* IR spectrometer equipped with a fiber–optical immersion probe using diamond as the ATR element. The conversion of DHA to GA and FA was carried out in a three-necked round bottom flask containing 10 mg 1wt%MnO_2/Goethite catalyst, 1.0 mmol DHA, 100 uL 50wt% H_2O_2

and 1.5 mL D_2O. D_2O is used as solvent to avoid overlap of the δ(O–H) vibration of H_2O with the ν(C=O) vibrations of carbonyl compounds and a higher amount of 1wt%MnO_2/Goethite catalyst is used to overcome the sensitivity problem.

DMPO-trapped EPR procedures. The EPR experiments were performed in a round bottom flask containing 10 mg catalyst, 1.0 mmol DHA, 100 uL 50wt% H_2O_2, and 1.5 mL D_2O (Eq. 1). 50 μL reaction solutions were withdrawn and immediately mixed with 10 μL DMPO. The mixed solution was transferred to a glass microcapillary tube (Hirschmann). Then, the capillary tube was placed into a quartz EPR tube (φ4×250mm), and EPR spectra were recorded immediately at room temperature. All spectra were obtained under the following conditions: central field = 3430 G; sweep width = 100 G; sweep time =30 s; microwave frequency = 9.63729 GHz; microwave power = 0.3162 mW. Signal fitting was carried out by using the Spin Fit program (Bruker).

Quantitative analysis of HO· generation. The time-dependent generation rates of HO· in our near solvent-free system was determined using formic acid (FA) that is miscible with water in any ratio as the probe molecule. The loss rate of FA follows pseudo-first-order kinetics and can be calculated as:

$$-\frac{d[\mathrm{FA}]}{dt} = k_{\mathrm{FA}}[\mathrm{FA}] = k_{\mathrm{HO\cdot,FA}}[\mathrm{HO\cdot}]_{\mathrm{ss}}[\mathrm{FA}] \quad (1)$$

$$[\mathrm{HO\cdot}]_{\mathrm{ss}} = \frac{k_{\mathrm{FA}}}{k_{\mathrm{HO\cdot,FA}}} \quad (2)$$

where k_{FA} is the observed rate constant (h^{-1}) that can be calculated via nonlinear least-squares fitting of the kinetic data of FA transformation, and $k_{\mathrm{HO\cdot,FA}}$ is the second-order rate constant (7.92×10^{12} $M^{-1}h^{-1}$).[5] Since the scavenging effect of other species on HO· cannot be ruled out in the near solvent-free system, the H_2O_2 and released Mn^{2+} are also included as primary scavengers. Thus, the generation rate of HO· at steady state is described as the following equation:

$$\begin{aligned} V_{\mathrm{HO\cdot}} &= \sum k_{\mathrm{HO\cdot,i}}[\mathrm{HO\cdot}]_{\mathrm{ss}}[\mathrm{S_i}] \\ &= k_{\mathrm{HO\cdot,FA}}[\mathrm{HO\cdot}]_{\mathrm{ss}}[\mathrm{FA}] + k_{\mathrm{HO\cdot,H_2O_2}}[\mathrm{HO\cdot}]_{\mathrm{ss}}[\mathrm{H_2O_2}] + k_{\mathrm{HO\cdot,Mn^{2+}}}[\mathrm{HO\cdot}]_{\mathrm{ss}}[\mathrm{Mn^{2+}}] \end{aligned} \quad (3)$$

where $k_{\mathrm{HO\cdot,H_2O_2}}$ and $k_{\mathrm{HO\cdot,Mn^{2+}}}$ are 9.72×10^{10} $M^{-1}h^{-1}$ and 1.224×10^{11} $M^{-1}h^{-1}$,

respectively.[5,6] The concentration of these species in bulk solution are used because the diffusion is fast compared to the rate of decomposition of these species.[5]

We calculated $V_{HO\cdot}$ via Supplementary Eq. 3. To avoid the scavenging effect of FA degradation products on HO·, FA was added at each recorded time point instead of the zero point of the reaction and the observed rate constant was calculated using kinetic data within the initial 1 hour of the reaction. The initial concentration of FA was 2 M for the first 4 time points (0 h, 1 h, 3 h, and 7 h) or 0.2 M for the last 2 time points (11 h and 23 h) in the MnO_2/Goe system, while the initial concentration of FA was kept at 0.2 M for all time points in the pure Goe system.

Calculation of the DHA conversion, product selectivity of GA and FA, carbon atom efficiency and oxygen atom efficiency. The conversion of DHA was calculated as:

$$\mathrm{Con} = \frac{n_{\mathrm{DHA}}}{n_{\mathrm{DHA,initial}}} \times 100\ \% \tag{4}$$

where Con (%) is the conversion of DHA, $n_{\mathrm{DHA,initial}}$ and n_{DHA} (mmol) is the mol of the initial and final DHA detected. The selectivity to a specific product was calculated as:

$$\mathrm{Sel} = \frac{n_{\mathrm{product}}}{n_{\mathrm{DHA,initial}} - n_{\mathrm{DHA}}} \times 100\ \% \tag{5}$$

where Sel (%) is the selectivity, n_{product}, $n_{\mathrm{DHA,initial}}$ and n_{DHA} (mmol) is the mol of the specific product , initial and final DHA detected. The yield to a specific product was calculated as:

$$Y = \frac{\mathrm{Con}}{100} \times \frac{\mathrm{Sel}}{100} \times 100\ \% \tag{6}$$

where Y (%) is the yield. The carbon atom efficiency was calculated by the following formula:

$$E_n = \frac{\sum a_i \times n_i}{3 \times n_{\mathrm{DHA,initial}}} \times 100\ \% \tag{7}$$

where E_n (%) is the carbon atom efficiency, a_i is the number of carbon atoms in the product molecule, n_i (mmol) is the mol of the generated product, $n_{\mathrm{DHA,initial}}$ (mmol) is the initial mol of the added DHA. The atom economy was calculated by the following formula:

$$\mathrm{AE} = \frac{n_{\mathrm{GA,initial}} \times \mathrm{MW_{GA}} + n_{\mathrm{FA,initial}} \times \mathrm{MW_{FA}}}{n_{\mathrm{DHA,initial}} \times \mathrm{MW_{DHA}} + 2 \times n_{\mathrm{H_2O_2,initial}} \times \mathrm{MW_{H_2O_2}}} \times 100\ \% \quad (8)$$

where AE (%) is the atom economy, MW is the molecular weight of the specific compound. The oxygen atom utilization efficiency was calculated by the following formula:

$$\mathrm{OE} = \frac{\sum n_i \times N_{\mathrm{oxygen,i}}}{2 \times n_{\mathrm{H_2O_2,initial}}} \times 100\ \% \quad (9)$$

where OE (%) is the oxygen atom utilization efficiency, n_i (mmol) is the mol of the generated product, $N_{\mathrm{oxygen,i}}$ is the number of oxygen atoms that need to be transferred from H_2O_2 when producing 1 unit of the product. $n_{\mathrm{DHA,initial}}$ (mmol) is the initial mol of the added H_2O_2.

Calculation of the ratio of protonated/unprotonated FA and average intrinsic rate constants of FA with HO·. The ratio of protonated/unprotonated FA was first calculated by the following equation:

$$\mathrm{pH} = \mathrm{pKa_{FA}} + \lg \frac{[\mathrm{HCO_2^-}]}{[\mathrm{HCO_2H}]} \quad (10)$$

where $[HCO_2^-]$, $[HCO_2H]$ is the concentration of unprotonated and protonated FA, respectively, pH is the pH value of Cu/Al_2O_3 or 1%-MnO_2/Goe system (pKa_{FA}=3.55). The average intrinsic rate constants of FA with HO· in the Cu/Al_2O_3 or 1%-MnO_2/Goe system was calculated as:

$$k_{\mathrm{FA,HO\cdot}} = k_{\mathrm{HCOOH,HO\cdot}} \times \frac{[\mathrm{HCOOH}]}{[\mathrm{HCOOH}] + [\mathrm{HCOO^-}]} + k_{\mathrm{HCOO^-,HO\cdot}} \times \frac{[\mathrm{HCOO^-}]}{[\mathrm{HCOOH}] + [\mathrm{HCOO^-}]}$$

(11)

where $k_{\mathrm{HCO_2^-,\,HO\cdot}}$ =3.2×10^9 M^{-1}s^{-1}, $k_{\mathrm{HCO_2H,\,HO\cdot}}$ =1.3×10^8 M^{-1}s^{-1}.

Supplementary Tables

Supplementary Table 1. Iron leaching of different iron (hydro)oxides.

**Iron (hydro)oxides**	**α-Fe_2O_3**	**FeS_2**	**Fe_3O_4**	**γ-FeOOH**	**α-FeOOH**
**Iron leaching (%)**	0.04	91.46	2.88	0.97	0.09

Supplementary Table 2. Comparison of oxygen atom utilization efficiency for the typical heterogeneous catalytic oxidation processes using H_2O_2 as oxidant. *

Entry	Catalyst	Substrate	Product	Reaction condition	Catalytic performance	Oxygen atom utilization efficiency	Ref.
**1**	Fe-CN/TS-1	benzene	phenol	benzene (9 mmol), H_2O_2 (5 mmol), MeCN (4 mL), water (4 mL), catalyst (50 mg), 300 W Xenon lamp (λ ≥ 420 nm), 60°C, 4 h	yield: 10%	9%	7
**2**	FeN4/GN	benzene	phenol	benzene (4.5 mmol), H_2O_2 (200 mmol), MeCN (3 mL), catalyst (50 mg), 25°C, 24 h	yield: 23.4%	0.26%	8
**3**	MIL-100(Fe)	benzene	phenol	benzene (0.5 mmol), H_2O_2 (0.375 mmol), catalyst (10 mg), solvent (4 mL), light irradiation (λ ≥ 420 nm), 24 h	conversion: 30.6% selectivity: 98%	20.0%	9
**4**	FeOCl	benzene	phenol	benzene (10 mmol), H_2O_2 (10 mmol), catalyst (0.1 g), acetic acid (1 mL), 60°C, 4 h	yield: 43.5%	21.8%	10

**5**	SA-Fe/CN	benzene	phenol	benzene (5.6 mmol), H_2O_2 (50.4 mmol), MeCN (6 mL), 60°C, 24 h	yield: 45%	2.5%	11
**6**	Fe@NC	benzene	phenol	benzene (2.5 mmol), H_2O_2 (20 mmol), MeCN (3 mL), water (3 mL), catalyst (30 mg), 60°C, 4 h	yield: ~17.5%	1.1%	12
**7**	$CuPdO_2/CuO$	methane	methanol	methane (11.2 mmol), H_2O_2 (66.5 mmol), catalyst (10 mg), 50°C, 1 h	yield: 0.041 mmol	0.031%	13
**8**	Fe/Fe_3C	methane	formic acid	methane (1 atm), H_2O_2 (500 mmol), catalyst (8 mg), H_2O(4 mL), 1500 W m^{-2} Xe lamp, 25°C, 4 h	yield: 0.037 mmol	0.0074%	14
**9**	Au/SiO_2	glycerol	acetic acid	glycerol (0.1 mmol) H_2O_2 (3.7 mmol), catalyst (20 mg), 80°C, 24 h	conversion: 100.0% selectivity: 90%	1.2%	15
**10**	SiO_2-MnO_2	glycerol	acrylic acid	glycerol (10 mmol), H_2O_2 (50 mmol), catalyst: 0.046 g, CH_3CN (10 mL), 70°C, 3 h	conversion: 77.1% selectivity: 74.7%	5.8%	16

**11**	Au/CuNiAlO	glycerol	glyceric acid	glycerol (10 mmol), H_2O_2 (22.05 mmol), catalyst: 0.2 g, 60°C, 4 h	conversion: 90.5% selectivity: 72.0%	14.8%	17
**12**	Cu/Al_2O_3	1,3-dihydroxyacetone	glycolic acid, formic acid	1,3-dihydroxyacetone (1 mmol), H_2O_2 (6 mmol), catalyst (25 mg), 25°C, 24 h	yield (glycolic acid): 94% yield (formic acid): 41%	11.2%	18
**13**	MnO_2/Goe	1,3-dihydroxyacetone	glycolic acid, formic acid	1,3-dihydroxyacetone (1 mmol), H_2O_2 (2 mmol), catalyst (30 μmol), 25°C, 24 h	yield (glycolic acid): 83.2% yield (formic acid): 93.4%	44.2% 46.6% (under O_2 pressure)	this study

*The oxygen atom utilization efficiency was calculated based on the reported data in the literatures.

Supplementary Table 3. Reaction conditions and catalytic performance of pure goethite and MnO_2/Goe catalysts for the C-C cleavage of other biomass-based platform molecules.

Substrate	Reaction conditions[a]	Catal.	Con. (%)	Products[b] (mmol)	C atom selectivity[c] (%)
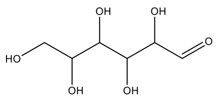	H_2O_2: 3.3 mmol<br>36 h	Goe	14.70	FA: 0.18<br>AA: 0.061<br>EA: 0.012	51.13
		MnO_2/Goe	18.10	FA: 0.26<br>AA: 0.087<br>EA: 0.058	85.36
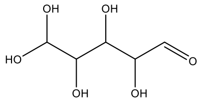	H_2O_2: 3.3 mmol<br>36 h	Goe	20.14	FA: 0.12<br>EA: 0.094<br>GLA: 0.018	54.62
		MnO_2/Goe	64.70	FA: 0.73<br>EA: 0.48<br>GLA: 0.023	92.40
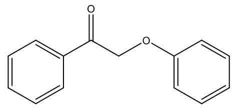	H_2O_2: 6 mmol<br>CH_3CN: 0.6 mL<br>48 h	Goe	6.88	FA: 0.009<br>BA: 0.058<br>Phen: 0.007<br>Cat: 0.008	52.43
		MnO_2/Goe	17.43	FA: 0.019<br>BA: 0.162<br>Phen: 0.071	64.71
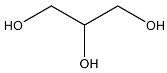	H_2O_2: 3.3 mmol<br>initial pH <2[d]<br>24 h	Goe	3.96	FA: 0.015<br>GA: 0.021<br>OA: 0.009	63.13
		MnO_2/Goe	19.87	FA: 0.17<br>GA: 0.11<br>OA: 0.042	79.52
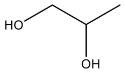	H_2O_2: 3.3 mmol<br>initial pH <2[d]<br>24 h	Goe	16.47	FA: 0.082<br>GA: 0.071<br>PL: 0.009	50.80
		MnO_2/Goe	29.11	FA: 0.23<br>GA: 0.21<br>PL:0.011	78.21

[a] Other condition: catalyst 30 μmol, substrate 1 mmol, 25°C, 400 rpm.

[b] AA: Arabic acid (C5H10O6) BA: Benzoic acid (C7H6O2) Cat: Catechol (C6H6O2) EA: Erythronic acid (C4H8O5) FA: Formic acid (CH2O2) GA: Glycolic acid (C2H4O3) GLA: Glyceric acid (C3H6O4) Phen: Phenol (C6H6O) PL: Pyruvaldehyde (C3H4O2) OA: Oxalic acid (C2H2O4)

[c] Unknown aromatic intermediates were detected in the conversion of 2-phenoxyacetophenone, but was not included in the quantification of carbon atom selectivity.

[d] pH adjusted using 20 wt% HNO_3 aqueous.

Supplementary Table 4. Reusability of the 1%-MnO_2/Goe catalyst.

Reuse cycles	1st	2nd	3rd	4th	5th
**GA Yields (%)**	83.2	78.7	79.8	77.3	79.4
**FA Yields (%)**	93.4	84.0	78.2	82.1	80.6
**Carbon atom efficiency (%)**	86.6	80.5	79.3	78.9	79.8
**Leached Fe (%)**	0.09	0.14	0.03	0.03	0.07
**Leached Mn (%)**	8.77	1.73	1.08	1.91	1.77

Supplementary Table 5. Catalytic performance of the 2500-times scale-up batch system.

DHA conversion (%)	GA Yield (%)	FA Yield (%)	Carbon atom efficiency (%)	Oxygen atom utilization efficiency (%)
89.4	80.9	92.7	84.8	42.7

Supplementary Table 6. Comparison of productivity for the valorization of DHA and other glycerol-based compounds.

Entry	Catalyst	Substrate	Product	Reaction condition	Productivity ($g_{product}$ $g_{cat.}^{-1}$ h^{-1})	Ref.
1	$FePO_4$ (continuous)	glycerol	acrolein	40 wt% of glycerol in water, 0.48 g/h, 280 °C	0.2	19
2	Pt/PVPc + TiO_2 (continuous)	glycerol	lactic acid	catalyst 50 mg, 5 mL aqueous glycerol solution (0.2 mol), O_2 0.5 MPa, 150 °C	0.1	20
3	Pt/ZSM-5 (continuous)	glycerol	propylene	catalyst 1 g, 59 wt% of glycerol in water, 250 °C	0.08	21
4	Sn-MFI (continuous)	glyoxal	glycolic acid	catalyst 269 mg, 2 wt% glyoxal aqueous solution, 0.3 mL/min, 90 °C	1.59	22
5	NbPO (continuous)	1,3-dihydroxyacetone	lactic acid	catalyst 200 mg, 0.4 M DHA aqueous solution, 0.2 mL/min, 150°C	0.78	23
4	Cu/Al_2O_3 (batch)	1,3-dihydroxyacetone	glycolic acid, formic acid	catalyst 25 mg, DHA 1 mmol, H_2O_2 6 mmol, 25 °C, 24 h	0.12 (glycolic acid) 0.03 (formic acid)	18
5	MnO_2/Goe (continuous)	1,3-dihydroxyacetone	glycolic acid, formic acid	catalyst 2g, 1 mmol DHA in 2 mmol H_2O_2 aqueous solution (10 wt%), 0.15 mL/min, 25 °C	0.35 (glycolic acid) 0.24 (formic acid)	this study

6	MnO_2/Goe (batch)	1,3-dihydroxyacetone	glycolic acid, formic acid	catalyst 75 mmol, DHA 2500 mmol, 30 wt% H_2O_2 500 mL, 25 °C, 24 h	0.96 (glycolic acid) 0.67 (formic acid)	

Supplementary Table 7. XPS Fe 2$p_{3/2}$ fitting results for used Goe and 1%-MnO_2/Goe samples.

Sample	Fe species	Binding Energy/ eV	FWHM /eV	Area	L-G/%
used Goe	Fe(II)	710.56	1.05	13240.01	0
	Fe(III)	711.61	2.79	98098.75	0
	Fe(II)$_{satellite}$	714.15	1.55	8826.67	0
used 1%-MnO_2/Goe	Fe(II)	710.64	1.38	19989.08	6
	Fe(III)	711.69	2.74	80495.75	0
	Fe(II)$_{satellite}$	714.09	1.70	12493.17	33

Supplementary Table 8. Kinetic behavior of all elemental steps for DHA oxidation.

non-HMOO pathway		HMOO pathway	
Equation	kinetic behavior	Equation	kinetic behavior
7	k_{ins}=10^8-10^{10} $M^{-1}s^{-1}$*	7	k_{ins}=10^8-10^{10} $M^{-1}s^{-1}$*
8	k_{ins}>10^9 $M^{-1}s^{-1}$*	12	k_{ins}=4.5*10^9 $M^{-1}s^{-1}$ [S24]
9	k_{ins}=10^9 $M^{-1}s^{-1}$ [S25]	13	k_{ins}=8.5*10^8 $M^{-1}s^{-1}$ [S26]<br>(HMOO formation)<br>k_{ins}=3.3*10^8 $M^{-1}s^{-1}$ [S26]<br>(HMOO formation)<br>Slow apparent kinetic<br>confirmed by ATR-IR
10	k_{ins}=3.5*10^9 $M^{-1}s^{-1}$ [S25]		
11	k_{ins}=1.4*10^9 $M^{-1}s^{-1}$ [S25]		

* The exact rate constants have not been reported in the literature until now and are speculated based on similar systems.[S27]

Supplementary Table 9. Structural information of all minerals.

	Samples	Phase	Sources	particle size	SSA (m^2/g)
Fe-bearing minerals	Hematite	α-Fe_2O_3	Commercial	<5 μm[a]	84.66
	Pyrite	FeS_2	Natural	>200 mesh[b]	11.61
	Magnetite	Fe_3O_4	Commercial	<5 μm[a]	42.78
	Lepidocrocite	γ-FeOOH	Commercial	<5 μm[a]	31.90
	Goethite	α-FeOOH	Commercial	>200 mesh[b]	17.53
	Goe-microrod	α-FeOOH	Synthesized	1~5 μm[b]	34.63
	Goe-nanorod	α-FeOOH	Synthesized	50-200 nm[c]	58.89
	Goe-CZ	α-FeOOH	Natural	>200 mesh[a]	5.01
	Goe-WS	α-FeOOH	Natural	>200 mesh[a]	7.44
	Mn/Goe	α-FeOOH	Synthesized	--	--
Mn-bearing minerals	Mn-rich mineral	SiO_2<br>$Mn_6O_{12}(H_2O)_{3.16}$<br>MnOOH<br>$M_{0.5}Mn_2O_3\cdot 1.5H_2O$	Natural	>200 mesh[a]	--
	Jacobsite	$MnFe_2O_4$	Commercial	--	--
	Hausmmannite	Mn_3O_4	Commercial	--	--
	Pyrolusite	β-MnO_2	Commercial	--	--

a. Obtained from the official website of vendor

b. After grinding
c. Observed by the SEM images

Supplementary Figures

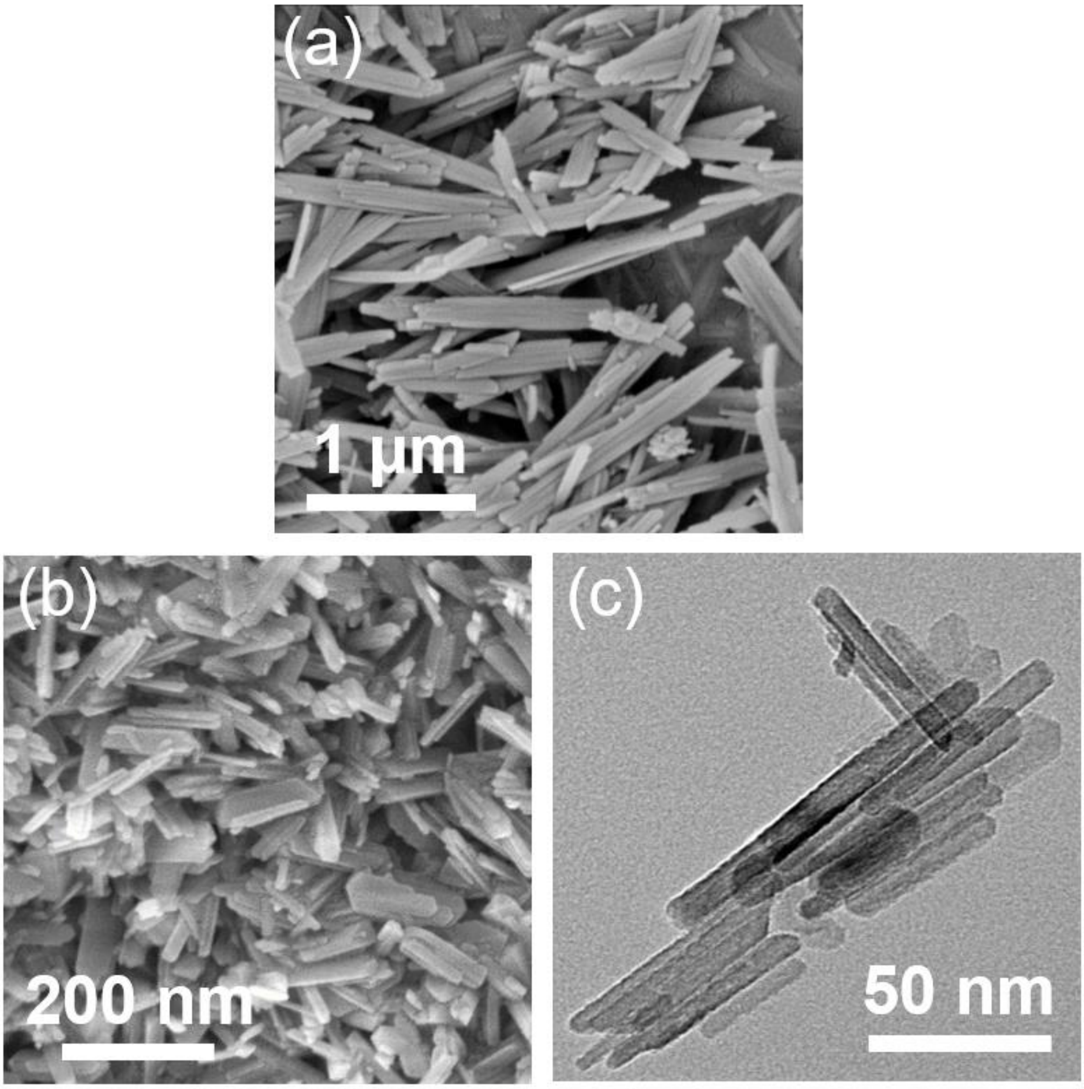

Supplementary Figure 1. SEM and TEM images of the goethite microrods (a) and nanorods (b-c).

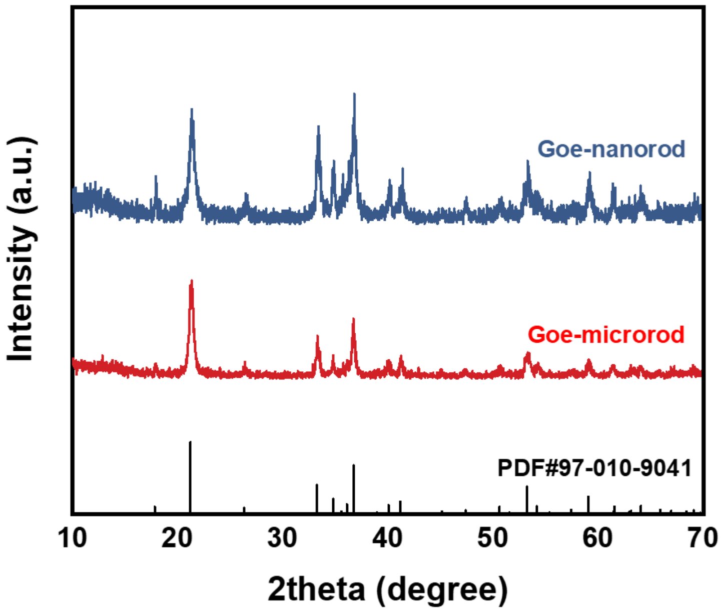

Supplementary Figure 2. XRD patterns of the goethite microrods and nanorods.

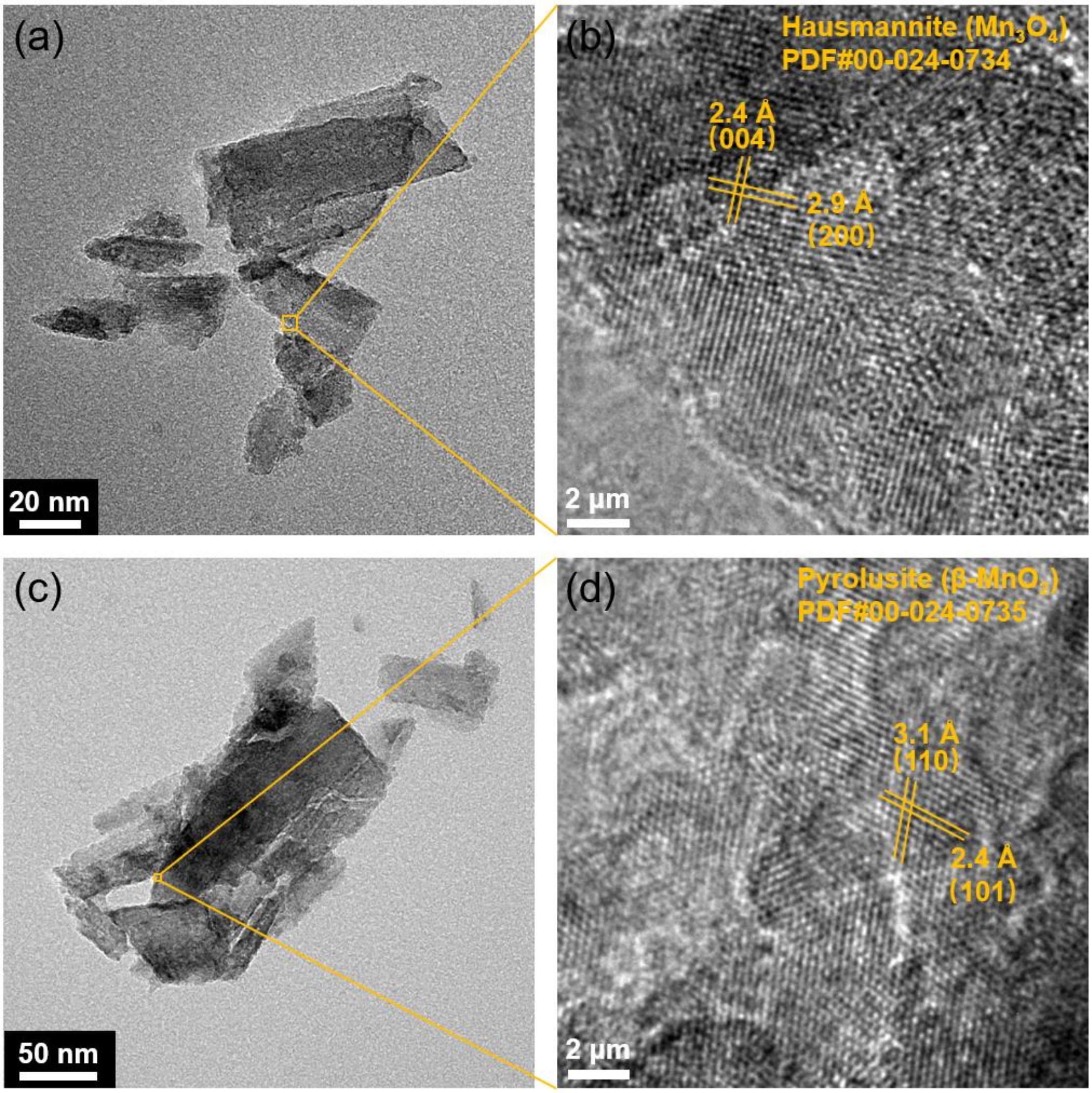

Supplementary Figure 3. TEM and HRTEM of the nanocrystalline MnO_x particles on Goe-CZ sample: hausmannite phase (a-b) and pyrolusite (c-d).

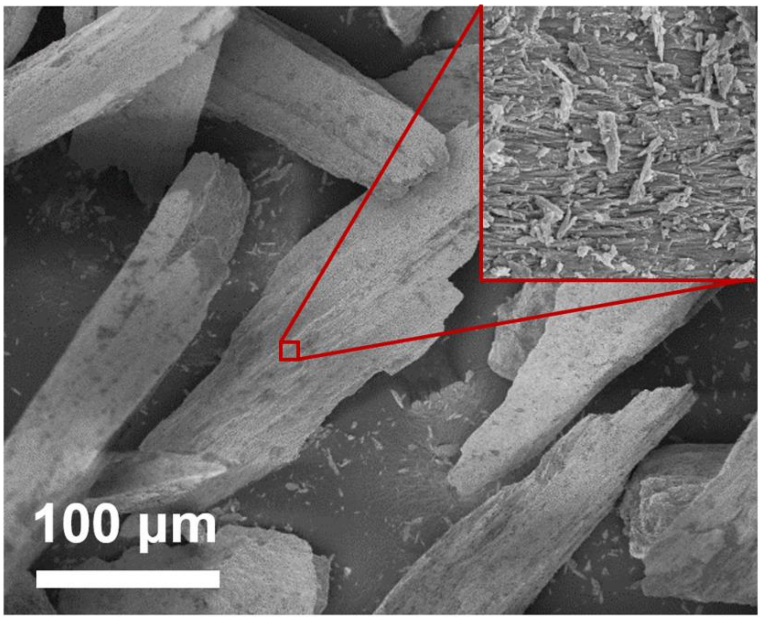

Supplementary Figure 4. SEM images of Goe-CZ.

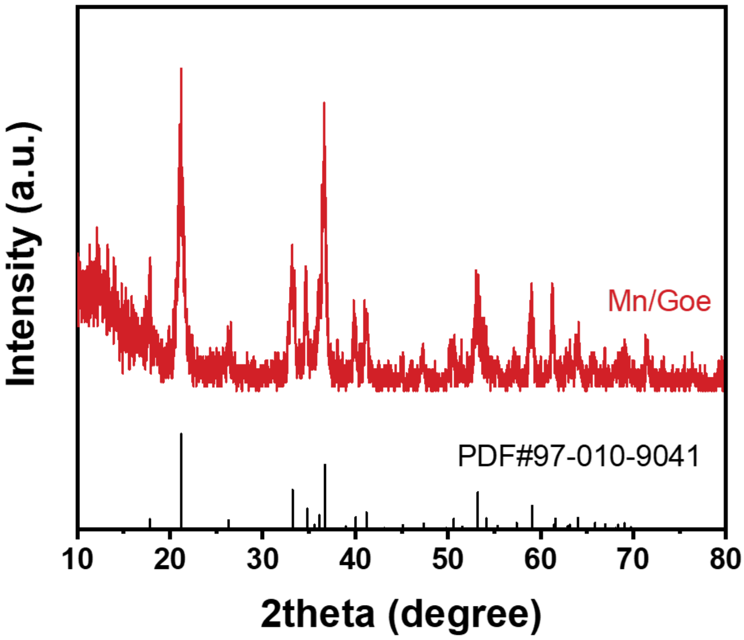

Supplementary Figure 5. XRD pattern of Mn/Goe catalyst.

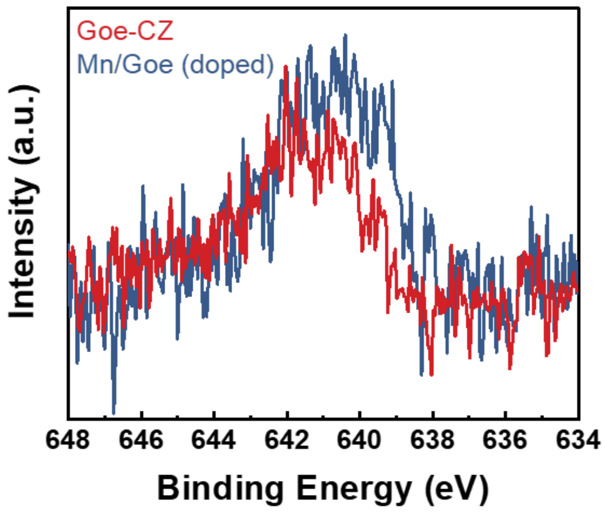

Supplementary Figure 6. Overlaid Mn 2$p_{3/2}$ spectra of Goe-CZ and Mn/Goe.

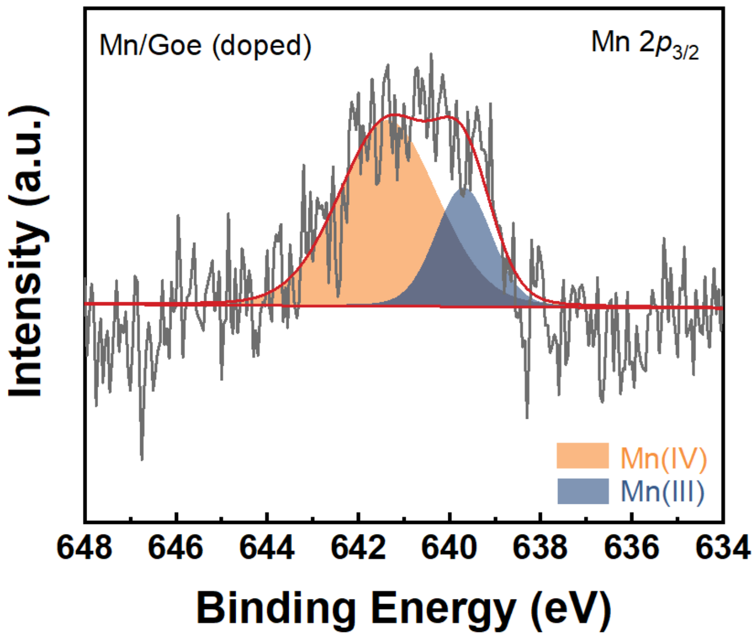

Supplementary Figure 7. Mn 2$p_{3/2}$ XPS spectra of Mn/Goe catalyst.

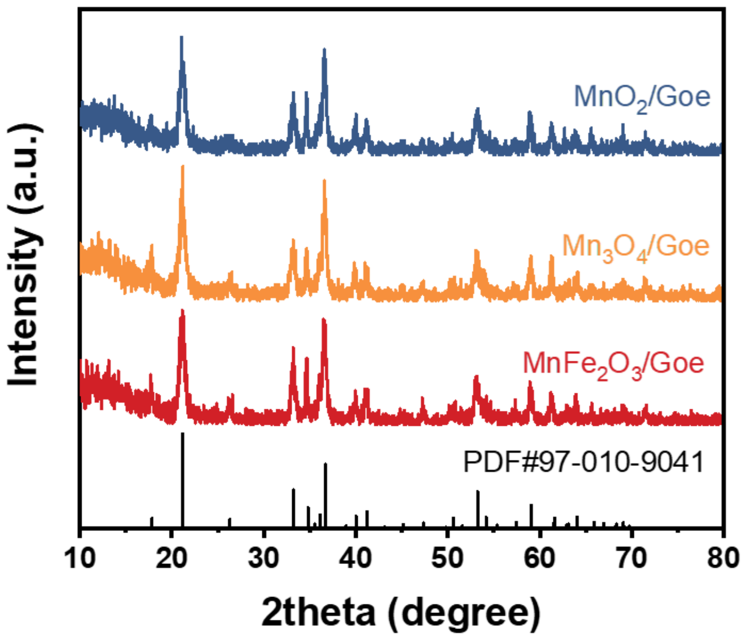

Supplementary Figure 8. XRD pattern of $MnFe_2O_3$/Goe, Mn_3O_4/Goe, MnO_2/Goe catalyst.

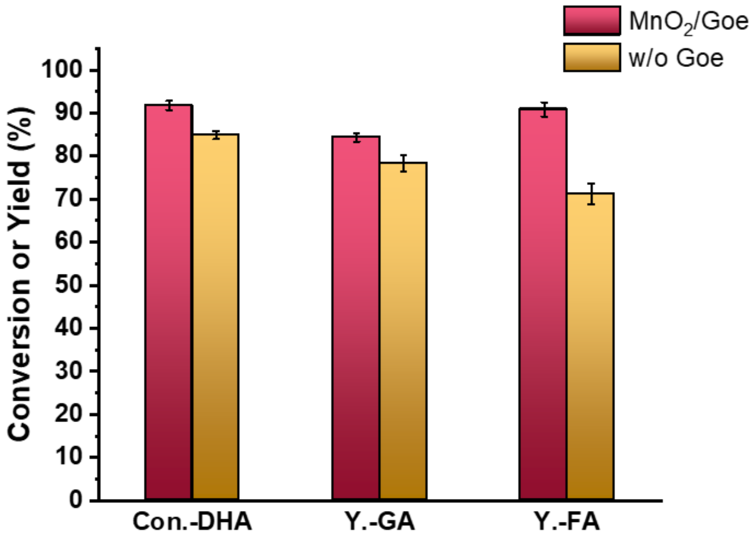

Supplementary Figure 9. Catalytic activity for DHA oxidation in the absence and presence of goethite. Reaction conditions: 1%-MnO_2/Goe 30 μmol (the mol of iron) or 1%-MnO_2/SiO_2 (with the equal mass of 1%-MnO_2/Goe), DHA 1 mmol, H_2O_2 0.2 mL (30 wt% H_2O_2 aqueous), 25°C, 400 rpm, 24 h.

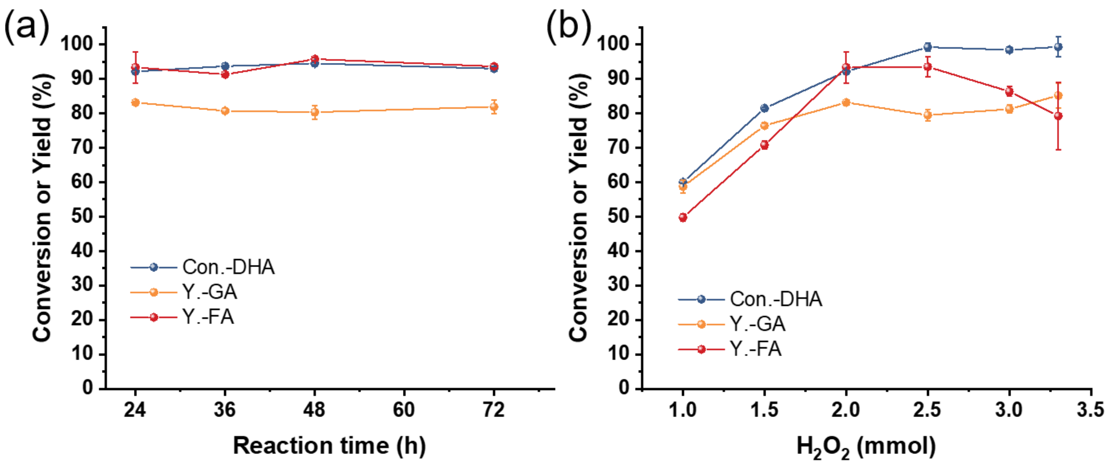

Supplementary Figure 10. Effect of reaction time (a) and H_2O_2 concentration (b) on the catalytic activity for DHA oxidation over MnO_2/Goe. Reaction conditions: 1%-MnO_2/Goe 30 μmol (the mol of iron), DHA 1 mmol, 25°C, 400 rpm, and (a) H_2O_2 0.2 mL (30 wt% H_2O_2 aqueous) and (b) 24 h.

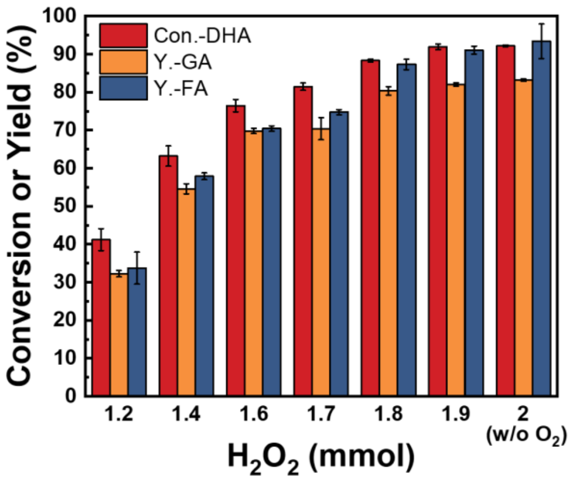

Supplementary Figure 11. Effect of H_2O_2 concentration on the catalytic activity for DHA oxidation over 1%-MnO_2/Goe under O_2 pressure. Reaction conditions: 1%-MnO_2/Goe 30 μmol, DHA 1 mmol, O_2 1.2 MPa, 25°C, 400 rpm, 24 h.

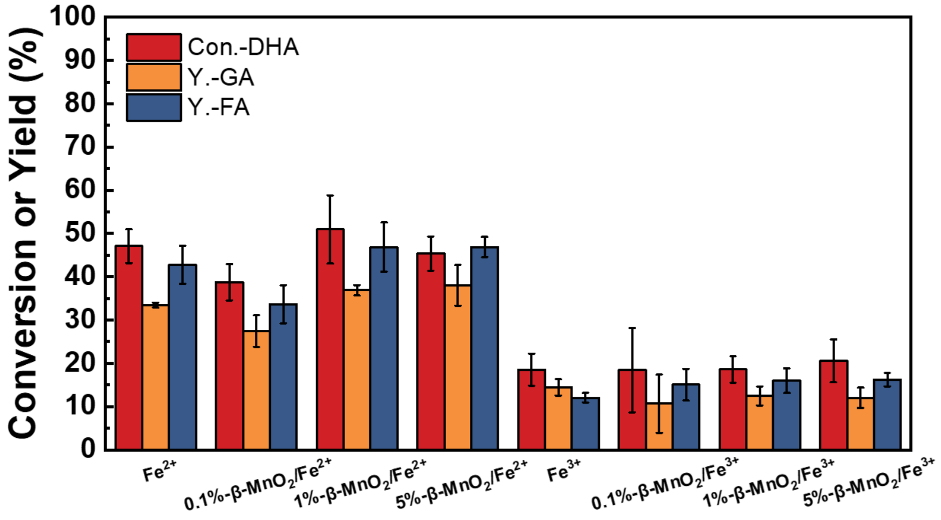

Supplementary Figure 12. Catalytic activity for DHA oxidation over MnO_2/iron ions ($FeSO_4$, $Fe(NO_3)_3$) catalysts with different mass ratio of Mn. Reaction conditions: catalyst 30 μmol, DHA 1 mmol, H_2O_2 0.2 mL (30 wt% H_2O_2 aqueous), 25°C, 400 rpm, 24 h.

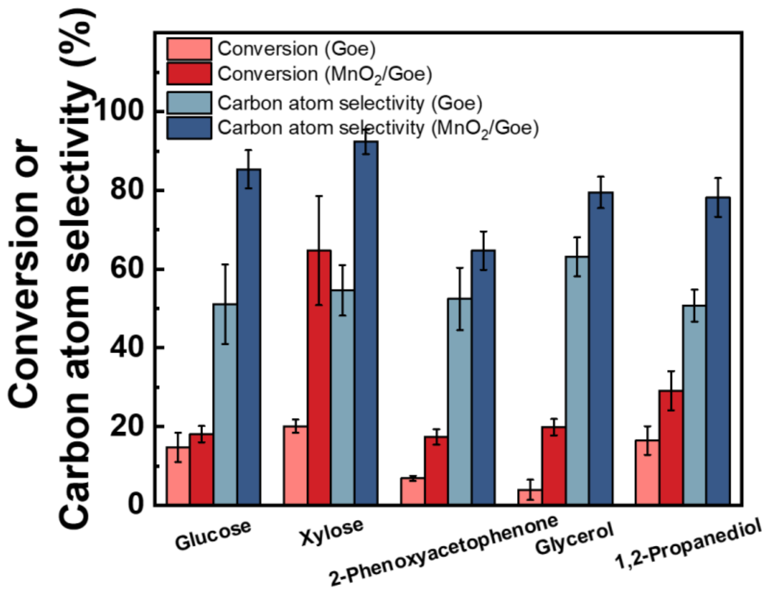

Supplementary Figure 13. Catalytic C-C cleavage of other biomass-based platform molecules. Reaction conditions were listed in Supplementary Table 3.

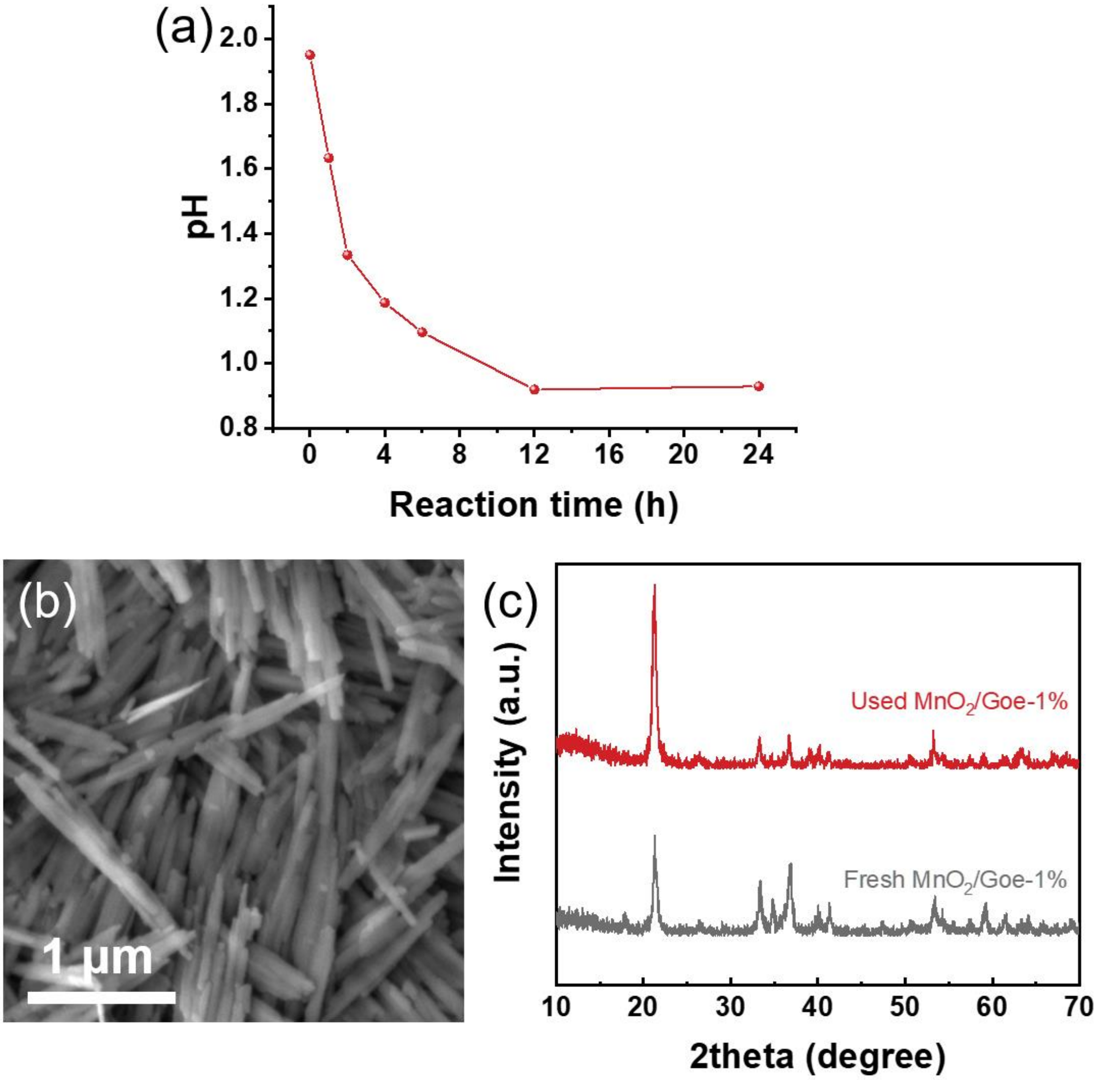

Supplementary Figure 14. Variation in pH during the reaction process (a). SEM image (b) and XRD pattern (c) of used 1%-MnO_2/Goe catalyst.

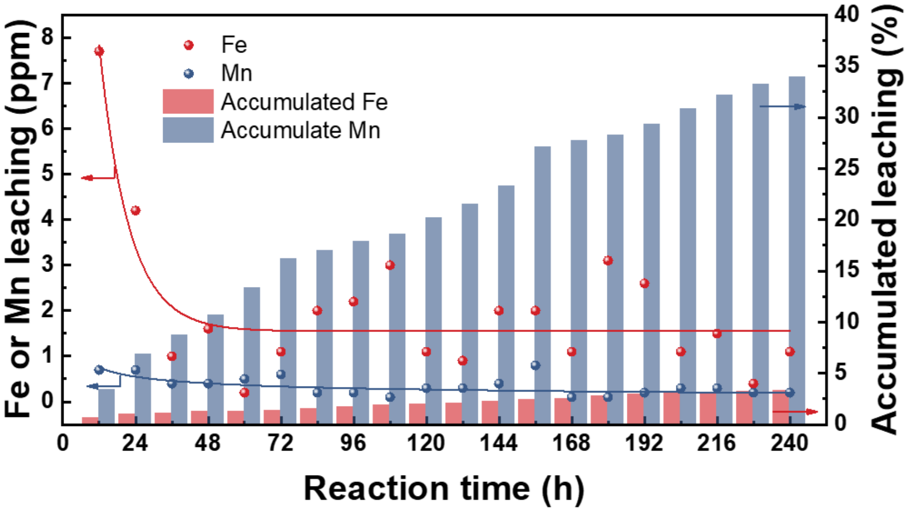

Supplementary Figure 15. Metal leaching of the MnO_2/Goe catalyst under the continuous flow test.

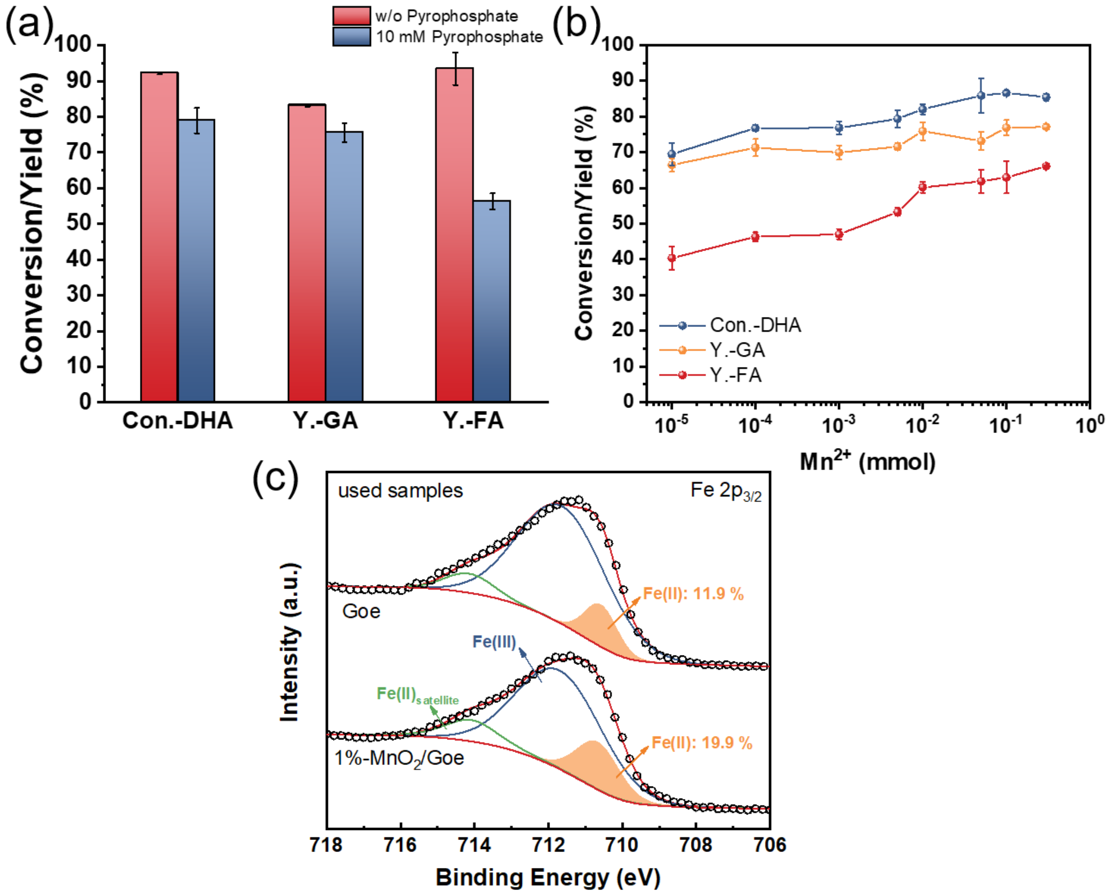

Supplementary Figure 16. Effect of pyrophosphate (a) and Mn^{2+} (b) on the catalytic activity for DHA oxidation over goethite. XPS spectra of Fe $2p_{3/2}$ for used goethite and 1%-MnO_2/Goe catalysts (c). Reaction conditions of pyrophosphate complexing experiment: 1%-MnO_2/Goe 30 μmol, DHA 1 mmol, H_2O_2 0.2 mL (30 wt% H_2O_2 aqueous containing 10 mM pyrophosphate, which was diluted with 50 wt% H_2O_2 aqueous), 25°C, 400 rpm, 24 h.

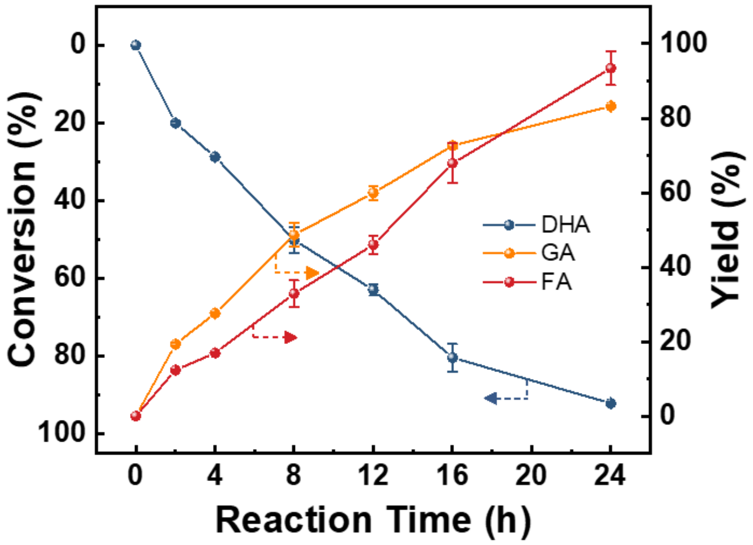

Supplementary Figure 17. DHA conversion and GA and FA yields profile over 1%-MnO_2/Goe catalyst as a function of time.

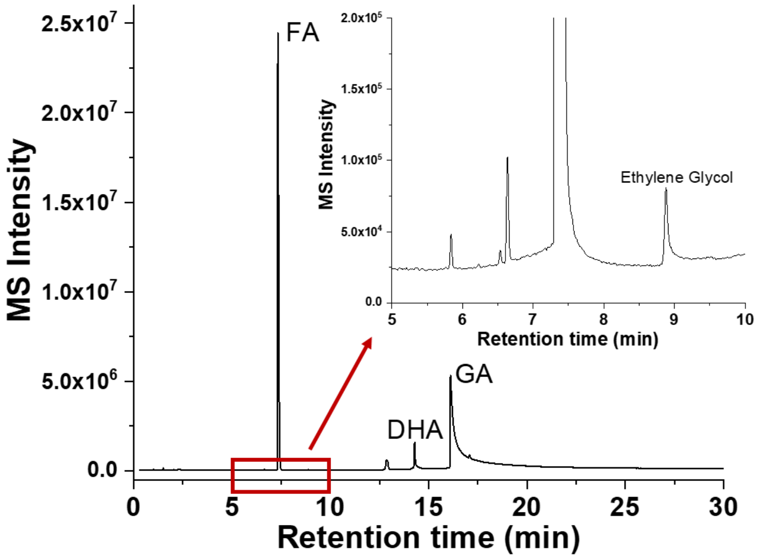

Supplementary Figure 18. GC-MS analysis of intermediates and products of DHA oxidation after reaction in the 1%-MnO_2/Goe system.

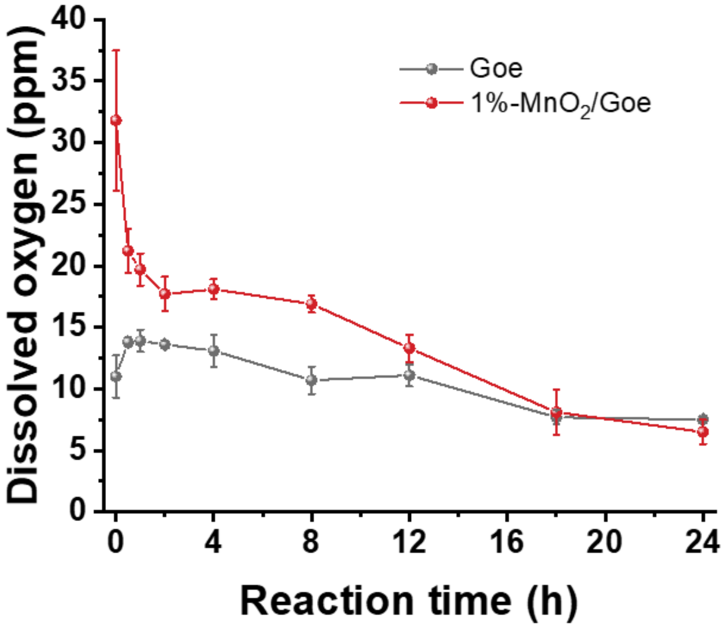

Supplementary Figure 19. Dissolved oxygen concentrations profile over pure Goe and 1%-MnO_2/Goe catalysts as a function of time.

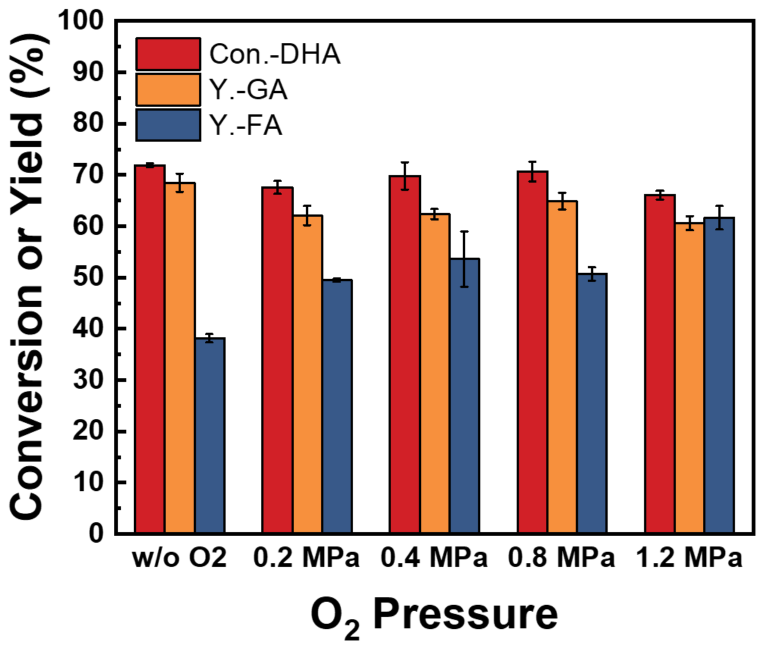

Supplementary Figure 20. Catalytic activity for DHA oxidation over pure goethite microrod under different O_2 pressure. Reaction conditions: catalyst 30 μmol, DHA 1 mmol, H_2O_2 0.2 mL (30 wt% H_2O_2 aqueous), 25°C, 400 rpm, 24 h.

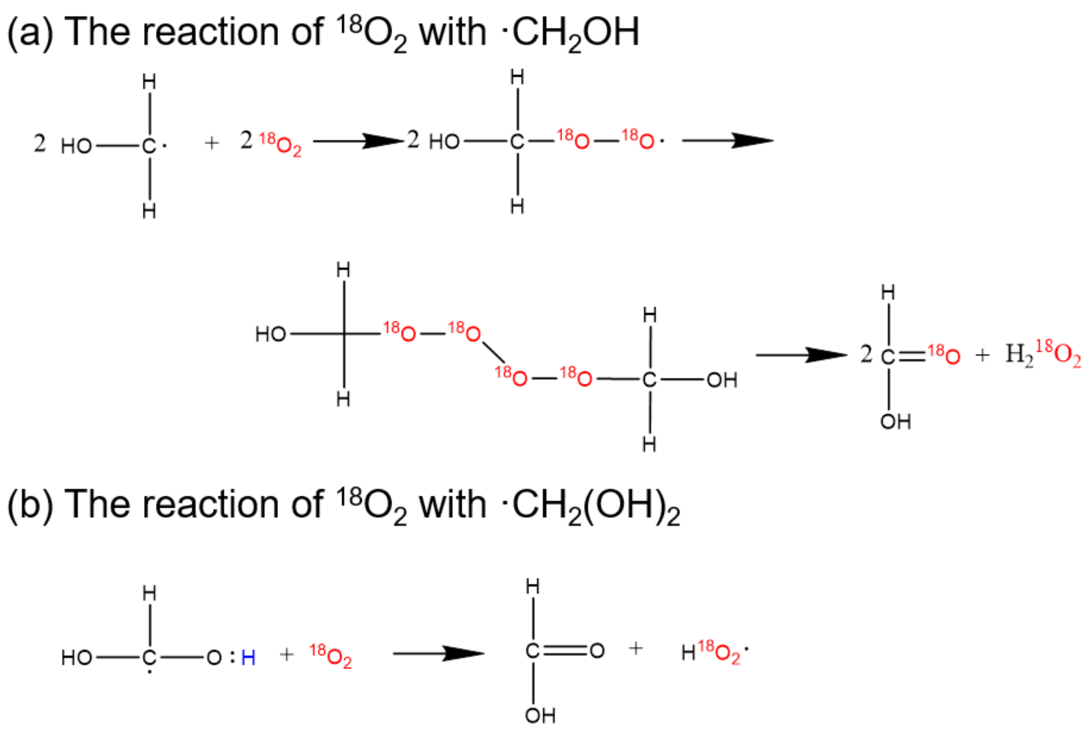

Supplementary Figure 21. Reaction pathways of $^{18}O_2$ with $\cdot CH_2OH$ (a) and $\cdot CH_2(OH)_2$ (b).

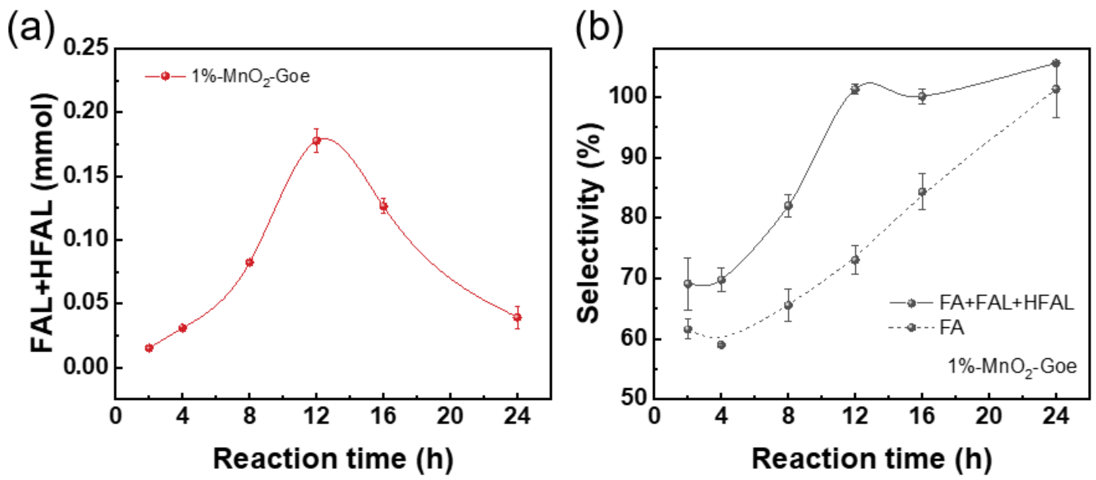

Supplementary Figure 22. Total concentrations profile of formaldehyde (FAL) and hydrated formaldehyde (HFAL) (a) and selectivity profile of all quantifiable products (b) over 1%-MnO_2/Goe catalyst as a function of time.

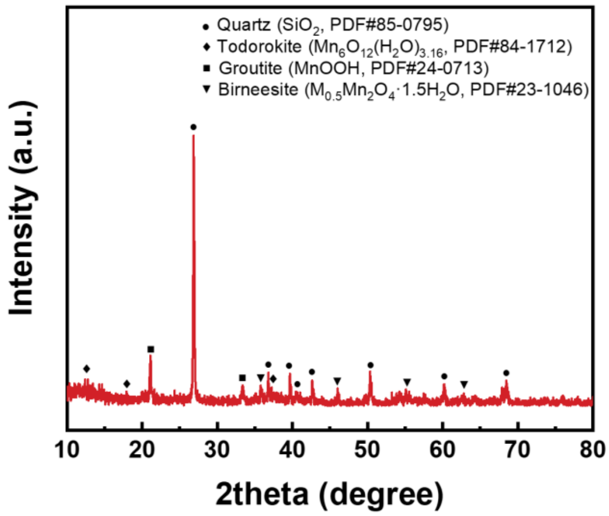

Supplementary Figure 23. XRD pattern of Mn-rich mineral.

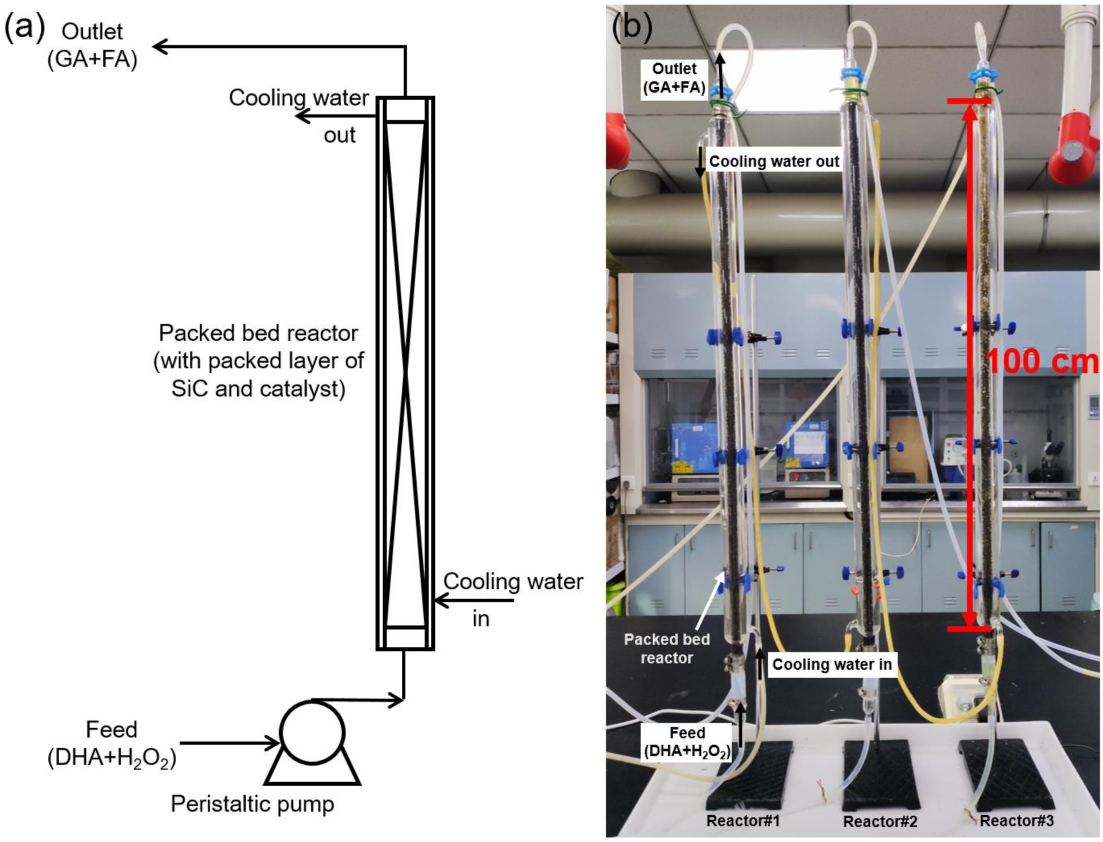

Supplementary Figure 24. Schematic of the continuous flow reaction process for DHA oxidation (a) and photograph of the packed bed reactor (three sets) (b).

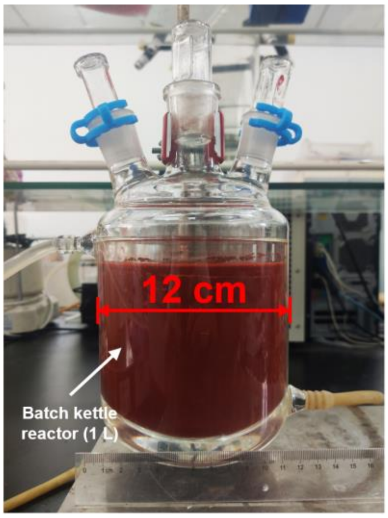

Supplementary Figure 25. Photograph of the 1L-batch kettle reactor.

Supplementary References

1 Schwertmann, U. & Cornell, R. M. *Iron oxides in the laboratory: preparation and characterization*. (John Wiley & Sons, 2008).

2 Anschutz, A. J. & Penn, R. L. Reduction of crystalline iron(III) oxyhydroxides using hydroquinone: Influence of phase and particle size. *Geochemical Transactions* **6**, 60 (2005).

3 Gerth, J. Unit-cell dimensions of pure and trace metal-associated goethites. *Geochim. Cosmochim. Acta* **54**, 363-371 (1990).

4 Hu, J., Lo & Chen, G. Fast Removal and Recovery of Cr(VI) Using Surface-Modified Jacobsite (MnFe2O4) Nanoparticles. *Langmuir* **21**, 11173-11179 (2005).

5 Kwan, W. P. & Voelker, B. M. Rates of Hydroxyl Radical Generation and Organic Compound Oxidation in Mineral-Catalyzed Fenton-like Systems. *Environmental Science & Technology* **37**, 1150-1158 (2003).

6 Jacob, D. J., Gottlieb, E. W. & Prather, M. J. Chemistry of a polluted cloudy boundary layer. *Journal of Geophysical Research: Atmospheres* **94**, 12975-13002 (1989).

7 Ye, X., Cui, Y., Qiu, X. & Wang, X. Selective oxidation of benzene to phenol by Fe-CN/TS-1 catalysts under visible light irradiation. *Applied Catalysis B: Environmental* **152-153**, 383-389 (2014).

8 Deng, D. *et al.* A single iron site confined in a graphene matrix for the catalytic oxidation of benzene at room temperature. *Science Advances* **1**, e1500462 (2015).

9 Wang, D., Wang, M. & Li, Z. Fe-Based Metal–Organic Frameworks for Highly Selective Photocatalytic Benzene Hydroxylation to Phenol. *ACS Catalysis* **5**, 6852-6857 (2015).

10 ElMetwally, A. E., Eshaq, G., Yehia, F. Z., Al-Sabagh, A. M. & Kegnæs, S. Iron Oxychloride as an Efficient Catalyst for Selective Hydroxylation of Benzene to Phenol. *ACS Catalysis* **8**, 10668-10675 (2018).

11 Zhang, M. *et al.* Metal (Hydr)oxides@Polymer Core–Shell Strategy to Metal Single-Atom Materials. *Journal of the American Chemical Society* **139**, 10976-10979 (2017).

12 Lu, E. *et al.* Selective Hydroxylation of Benzene to Phenol over Fe Nanoparticles Encapsulated within N-Doped Carbon Shells. *ACS Applied Nano Materials* **3**, 9192-9199 (2020).

13 Bai, S., Xu, Y., Wang, P., Shao, Q. & Huang, X. Activating and Converting CH4 to CH3OH via the CuPdO2/CuO Nanointerface. *ACS Catalysis* **9**, 6938-6944 (2019).

14 Xing, Y. *et al.* Fe/Fe3C Boosts H2O2 Utilization for Methane Conversion Overwhelming O2 Generation. *Angewandte Chemie International Edition* **n/a**.

15 Kapkowski, M. *et al.* SiO2-, Cu-, and Ni-supported Au nanoparticles for selective glycerol oxidation in the liquid phase. *Journal of Catalysis* **319**, 110-118 (2014).

16 Sarkar, B. *et al.* Cu nanoclusters supported on nanocrystalline SiO2–MnO2: a

bifunctional catalyst for the one-step conversion of glycerol to acrylic acid. *Chemical Communications* **50**, 9707-9710 (2014).

17 Wang, X., Wu, G., Jin, T., Xu, J. & Song, S. Selective Oxidation of Glycerol Using 3% H2O2 Catalyzed by Supported Nano-Au Catalysts. *Catalysts* **8**, 505 (2018).

18 Dai, X. *et al.* Sustainable Co-Synthesis of Glycolic Acid, Formamides and Formates from 1,3-Dihydroxyacetone by a Cu/Al2O3 Catalyst with a Single Active Sites. *Angewandte Chemie International Edition* **58**, 5251-5255 (2019).

19 Deleplanque, J., Dubois, J. L., Devaux, J. F. & Ueda, W. Production of acrolein and acrylic acid through dehydration and oxydehydration of glycerol with mixed oxide catalysts. *Catalysis Today* **157**, 351-358 (2010).

20 Komanoya, T. *et al.* A Combined Catalyst of Pt Nanoparticles and TiO2 with Water-Tolerant Lewis Acid Sites for One-Pot Conversion of Glycerol to Lactic Acid. *ChemCatChem* **8**, 1094-1099 (2016).

21 Wu, Z. *et al.* Selective Conversion of Glycerol into Propylene: Single-Step versus Tandem Process. *ACS Sustainable Chemistry & Engineering* **4**, 4192-4207 (2016).

22 Dapsens, P. Y., Mondelli, C., Kusema, B. T., Verel, R. & Pérez-Ramírez, J. A continuous process for glyoxal valorisation using tailored Lewis-acid zeolite catalysts. *Green Chemistry* **16**, 1176-1186 (2014).

23 Innocenti, G. *et al.* Continuous Liquid-Phase Upgrading of Dihydroxyacetone to Lactic Acid over Metal Phosphate Catalysts. *ACS Catalysis* **10**, 11936-11950 (2020).

24 Monod, A., Chebbi, A., Durand-Jolibois, R. & Carlier, P. Oxidation of methanol by hydroxyl radicals in aqueous solution under simulated cloud droplet conditions. *Atmospheric Environment* **34**, 5283-5294 (2000).

25 McElroy, W. J. & Waygood, S. J. Oxidation of formaldehyde by the hydroxyl radical in aqueous solution. *Journal of the Chemical Society, Faraday Transactions* **87**, 1513-1521 (1991).

26 Huie, R. E. & Clifton, C. L. Kinetics of the self-reaction of hydroxymethylperoxyl radicals. *Chemical Physics Letters* **205**, 163-167 (1993).

27 Buxton, G. V., Greenstock, C. L., Helman, W. P. & Ross, A. B. Critical Review of rate constants for reactions of hydrated electrons, hydrogen atoms and hydroxyl radicals (·OH/·O− in Aqueous Solution. *Journal of Physical and Chemical Reference Data* **17**, 513-886 (1988).
